# Machine Learning for Dynamic and Short-Term Prediction of Preeclampsia Using Routine Clinical Data

**DOI:** 10.1001/jamanetworkopen.2026.0359

**Published:** 2026-03-06

**Authors:** Haoyang Li, Yaxin Li, Chengxi Zang, Weishen Pan, He S. Yang, Tracy B. Grossman, Zhen Zhao, Fei Wang

**Affiliations:** 1 Department of Population Health Sciences, Weill Cornell Medicine, New York, New York; 2 Department of Pathology and Laboratory Medicine, Weill Cornell Medicine, New York, New York; 3 Division of Maternal-Fetal Medicine, NewYork-Presbyterian Brooklyn Methodist Hospital, Brooklyn, New York

## Abstract

**Importance:**

Preeclampsia is a leading cause of maternal and perinatal morbidity and mortality, yet its unpredictable onset and rapid progression hinder timely management. Existing prediction tools often rely on specialized biomarkers, static assessments, or limited study cohorts, impeding clinical utility and generalizability.

**Objective:**

To develop and validate machine learning models for dynamic, short-term prediction of preeclampsia onset using longitudinal electronic health record (EHR) data.

**Design, Setting, and Participants:**

This retrospective, multisite cohort study included pregnancies delivered between October 1, 2020, and May 31, 2025, at 3 NewYork-Presbyterian hospitals: Weill Cornell Medical College (WCMC), Lower Manhattan Hospital (LMH), and Brooklyn Methodist Hospital (BMH). Extreme gradient boosting models were developed to predict preeclampsia onset within 1, 2, and 4 weeks. Performance was assessed using nested cross-validation at the training site and external validation via direct transfer, fine-tuning, and retraining. The study included pregnancies among individuals 18 years or older (35 895 at WCMC, 8664 at LMH, and 14 280 at BMH).

**Exposure:**

Routine information captured within the EHR, including blood pressure, maternal characteristics and routine laboratory test results.

**Main Outcomes and Measures:**

The main outcome was development of preeclampsia within specified prediction windows. Model performance was evaluated using area under the receiver operating characteristic curve, specificity and positive predictive value at 90% sensitivity.

**Results:**

Among 58 839 pregnancies (mean [SD] maternal age, 33.3 [5.3] years; 10 196 [17.3%] Asian, 6525 [11.1%] Black or African American, 32 675 [55.5%] White, and 9443 [16.0%] other [ie, those who were races other than Asian, Black, or White] or unknown race), individuals who developed preeclampsia were older (median [IQR] age, 35.0 [31.0–38.0] vs 34.0 [31.0-37.0] years in the WCMC group [*P* < .001], 35.0 [31.0–38.0] years vs 34.0 [32.0-37.0] years in the LMH group [*P* = .003], and 33.0 [28.0–36.0] vs 31.0 [26.0–35.0] years in the BMH group [*P* < .001]) and more frequently Black (335 of 2227 [15.0%] vs 2178 of 3668 [6.5%] in the WCMC group [*P* < .001], 117 of 792 [14.8%] vs 566 of 7872 [7.2%] in the LMH group [*P* < .001], and 455 of 1088 [41.8%] vs 2874 of 13,192 [21.8%] in the BMH group [*P* < .001]). Predictive performance increased from 28 to 34 weeks’ gestation and peaked at 34 weeks’ gestation (areas under the receiver operating characteristic curves, 0.863 at training and 0.808-0.834 at validation). The positive predictive values increased from approximately 0.001 to 0.002 at 28 weeks to peak values at 36 weeks (mean [SD], 0.057 [0.012] at LMH and 0.046 [0.007] at BMH), whereas the negative predictive values were greater than 0.993. Blood pressure was the most informative predictor, whereas laboratory measures (including albumin, alkaline phosphatase, and hematologic indexes) contributed to earlier gestation, with demographic and obstetric factors increasing in importance later.

**Conclusions and Relevance:**

In this retrospective, multisite cohort study of pregnancies in late gestation, dynamic short-term prediction of preeclampsia was feasible using routinely available clinical and laboratory data. These results suggest that this approach provided opportunities for earlier intervention and would be adaptable across diverse health care settings.

## Key Points

**Question:**

Can dynamic, short-term prediction of preeclampsia in late gestation be achieved using routine data from electronic health records?

**Findings:**

In this cohort study of 58 839 pregnancies delivered at 3 NewYork-Presbyterian hospitals, prediction performance peaked at 34 weeks, demonstrating that preeclampsia in late gestation can be dynamically predicted with routinely available features.

**Meaning:**

This study’s results suggest that dynamic short-term prediction of preeclampsia using routine clinical data is feasible and provides actionable lead time for timely intervention in diverse health care settings.

## Teaser

This cohort study examines whether dynamic, short-term prediction of preeclampsia in late gestation could be achieved using electronic health record–derived clinical and laboratory data across multiple health care settings.

## Introduction

Preeclampsia is a hypertensive disorder of pregnancy that affects 2% to 8% of pregnancies worldwide and remains a leading cause of maternal and perinatal morbidity and mortality.^1,2,3^ Beyond direct health consequences, preeclampsia drives substantial health care utilization through preterm deliveries, neonatal intensive care admissions, and long-term follow-up for both mothers and infants.^4,5,6^ Its unpredictable onset and rapid progression pose critical challenges for obstetric care, making timely risk prediction a major unmet need.

A wide range of prediction strategies have been proposed, including maternal risk–based algorithms, biomarker panels, and machine learning models.^7,8,9,10^ However, important limitations constrain their utility and implementation. Many approaches focus on preeclampsia prediction in early pregnancy (first trimester) to guide aspirin prophylaxis and enhanced surveillance, yet both predictive accuracy and preventive efficacy have shown only modest and inconsistent results across studies.^11,12^ Others rely on specialized serum or ultrasound markers,^13,14,15,16^ increasing cost and reducing accessibility in routine or resource-limited settings. Generalizability is also limited because most models were developed in a single cohort, even though the prevalence and severity of preeclampsia vary across racial and ethnic groups,^17,18^ raising concerns of suboptimal performance when applied to different populations. Moreover, most existing tools assess risk at a specific time point and treat the risk statically rather than dynamically updating the risk as pregnancy progresses. Collectively, these limitations have hindered broad clinical adoption.

The timing of prediction is an underexplored dimension. Pregnancy is marked by dynamic physiologic changes, and preeclampsia can emerge abruptly, often in late gestation. Although routine prenatal visits generate longitudinal clinical and laboratory data, most existing models predict only whether preeclampsia will occur at some stage of pregnancy, without estimating when onset is short term.^8,10,19,20^ Approaches explicitly designed around rolling observation and prediction windows can better capture short-term risk trajectories and provide actionable lead time for surveillance or intervention.

We therefore examined whether dynamic, short-term prediction of preeclampsia in late gestation could be achieved using only routinely available electronic health record (EHR)–derived clinical and laboratory data across multiple health care settings. We hypothesized that machine learning models incorporating longitudinal EHR data could accurately predict preeclampsia onset within 1, 2, and 4 weeks and demonstrate consistent performance across demographically distinct hospital populations.

## Methods

### Data and Cohort

This retrospective, multisite cohort study was approved by the Weill Cornell Medicine institutional review board with a waiver of informed consent for secondary use of deidentified EHR data. Reporting followed the Strengthening the Reporting of Observational Studies in Epidemiology (STROBE) reporting guideline and Transparent Reporting of a Multivariable Prediction Model for Individual Prognosis or Diagnosis (TRIPOD) reporting guidelines.^21^ We included pregnancies delivered between October 1, 2020, and May 31, 2025, at 3 NewYork-Presbyterian hospitals: Weill Cornell Medical College (WCMC), Lower Manhattan Hospital (LMH), and Brooklyn Methodist Hospital (BMH). Eligible pregnancies involved individuals 18 years or older with documented delivery date and gestational age (GA). The final cohort included 58 839 pregnancies. Preeclampsia was identified using *International Statistical Classification of Diseases and Related Health Problems, Tenth Revision (ICD-10)* codes for antepartum preeclampsia (O11.x, O14.x), with onset defined as the admission date of the first qualifying diagnosis. Cohort construction details are in the eMethods in Supplement 1.

### Feature Extraction and Preprocessing

Model inputs consisted of routinely available EHR data, including demographic and obstetric characteristics, blood pressure (BP) measurements, and laboratory test results. Demographic and obstetric variables were treated as fixed covariates; BP was summarized as the median (IQR) within each observation window, and laboratory values reflected the most recent measurement. Features were selected based on clinical relevance and routine availability. Self-reported race data were collected to evaluate potential differences in model performance across racial groups. Race categories included Asian, Black or African American, White, and other [ie, those who were races other than Asian, Black, or White] or unknown race. Categorical variables were one-hot encoded. Continuous variables were median imputed and *z* standardized within training folds to prevent information leakage.^22^ Full feature definitions and preprocessing steps are described in the eMethods and eTable 1 in Supplement 1.

### Experimental Setting

We formulated preeclampsia prediction as a GA-anchored binary classification task using rolling observation and prediction windows. For each pregnancy, clinical data up to a specified observation week were used to predict preeclampsia onset within a subsequent prediction window of 1, 2, or 4 weeks. Labels were assigned based on preeclampsia occurrence within the prediction window.

### Model Development and Validation

We implemented extreme gradient boosting (XGBoost) as the primary modeling framework.^23^ Models were trained using nested stratified 5-fold cross-validation with class-weighted loss functions. Logistic regression and random forest models were evaluated as comparators. Model generalizability was assessed through in-site testing, direct transfer, fine-tuning, and retraining across WCMC, LMH, and BMH. Additional methodological details are given in the eMethods in Supplement 1.

### Statistical Analysis

#### Main Analysis

For cohort characteristics (Table 1), *P* values indicate group-wise comparisons between preeclampsia and controls within each site. For categorical variables, *P* values were computed using a χ^2^ test of independence based on contingency tables. For continuous variables, comparisons were made using a 2-sided Mann-Whitney *U* test. For model performance comparisons, statistical significance of differences was evaluated against the default XGBoost model using the Welch *t* test. A 2-sided *P* < .05 was considered statistically significant.

**Table 1.** Cohort Demographic and Baseline Characteristics

Characteristic	No. (%) of participants^a^								
	WCMC (n = 35 895)			LMH (n = 8664)			BMH (n = 14 280)		
	Preeclampsia (n = 2227)	Control (n = 33 668)	*P* value^b^	Preeclampsia (n = 792)	Control (n = 7872)	*P* value^b^	Preeclampsia (n = 1088)	Control (n = 13 192)	*P* value^b^
Maternal age at delivery, median (IQR), y	35.0 (31.0-38.0)	34.0 (31.0-37.0)	<.001	35.0 (31.0-38.0)	34.0 (32.0-37.0)	.003	33.0 (28.0-36.0)	31.0 (26.0-35.0)	<.001
Race^c^									
Asian	344 (15.4)	5659 (16.8)	.10	264 (33.3)	3336 (42.4)	<.001	34 (3.1)	559 (4.2)	.09
Black or African American	335 (15.0)	2178 (6.5)	<.001	117 (14.8)	566 (7.2)	<.001	455 (41.8)	2874 (21.8)	<.001
White	1077 (48.4)	20 424 (60.7)	<.001	254 (32.1)	2724 (34.6)	.16	408 (37.5)	7788 (59.0)	<.001
Other	308 (13.8)	2917 (8.7)	<.001	100 (12.6)	704 (8.9)	<.001	131 (12.0)	1209 (9.2)	.002
Unknown	160 (7.2)	2467 (7.3)	.84	56 (7.1)	538 (6.8)	.86	58 (5.3)	755 (5.7)	.64
Smoker									
Yes	242 (10.9)	2605 (7.7)	<.001	89 (11.2)	742 (9.4)	.11	64 (5.9)	597 (4.5)	.049
No	1972 (88.5)	30 743 (91.3)	<.001	697 (88.0)	7081 (90.0)	.10	1016 (93.4)	12 476 (94.6)	.11
Alcohol									
Yes	20 793 (61.8)	1399 (62.8)	.33	4657 (59.2)	498 (62.9)	.046	4119 (31.2)	377 (34.7)	.02
No	7562 (22.5)	543 (24.4)	.04	1968 (25.0)	178 (22.5)	.13	5070 (38.4)	411 (37.8)	.69
Multifetal gestation	172 (7.7)	705 (2.1)	<.001	45 (5.7)	176 (2.2)	<.001	44 (4.0)	265 (2.0)	<.001
Delivery GA, median (IQR), wk	37.6 (36.6-39.1)	39.3 (38.6-40.0)	<.001	38.1 (37.1-39.4)	39.3 (38.6-39.9)	<.001	37.7 (36.7-39.3)	39.6 (38.7-40.4)	<.001
Cesarean delivery	1231 (55.3)	10 853 (32.2)	<.001	348 (43.9)	2275 (28.9)	<.001	551 (50.6)	3229 (24.5)	<.001
Infant weight, median (IQR), g	2959.7 (2520.3-3365.1)	3280.0 (2973.8-3580.5)	<.001	3050.4 (2656.3-3424.6)	3246.2 (2959.6-3523.8)	<.001	2979.5 (2489.0-3418.9)	3322.5 (2999.3-3640.0)	<.001
Nulliparity	1524 (68.4)	18 390 (54.6)	<.001	583 (73.6)	4439 (56.4%)	<.001	600 (55.1)	5239 (39.7)	<.001
Pregravid BMI, median (IQR)	28.6 (25.3-32.9)	26.2 (23.9-29.4)	<.001	28.3 (25.0-32.1)	26.3 (24.0-29.5)	<.001	32.6 (28.1-37.7)	28.6 (25.4-32.9)	<.001

Abbreviations: BMH, Brooklyn Methodist Hospital; BMI, body mass index (calculated as weight in kilograms divided by the square of height in meters); GA, gestational age; LMH, Lower Manhattan Hospital; WCMC, Weill Cornell Medical College.

^a^ Unless otherwise indicated.

^b^ *P* values indicate group-wise comparisons between preeclampsia and controls within each site. For categorical variables, *P* values were computed using a χ^2^ test of independence based on contingency tables. For continuous variables, comparisons were made using a 2-sided Mann-Whitney *U* test.

^c^ Race was self-reported by participants and recorded in the electronic health record. Other includes participants who did not identify as Asian, Black, or White, and unknown includes those who declined to report race.

Model interpretability was assessed using Shapley Additive Explanations (SHAP). Feature importance was summarized using mean absolute SHAP values derived from held-out test folds. Feature contributions were compared between earlier and later gestational prediction tasks. Performance was evaluated using the area under the receiver operating characteristic curve (AUC), area under the precision-recall curve (AUPRC), specificity, positive predictive value (PPV) and negative predictive value (NPV) at 90% sensitivity, and the Brier score. Metrics were summarized across GAs, prediction windows, and validation strategies.

#### Sensitivity Analyses

Sensitivity analyses assessed model robustness to feature exclusion (eg, BP or laboratory tests), alternative algorithms, and additional modeling choices. A Cox proportional hazards regression model was also evaluated for time-to-event prediction of preeclampsia onset. Detailed results and methods are given in the eMethods in Supplement 1.

## Results

### Cohort Characteristics

The study cohort was constructed from all pregnant individuals aged 18 years or older (mean [SD] maternal age, 33.3 [5.3] years; 10 196 [17.3%] Asian, 6525 [11.1%] Black or African American, 32 675 [55.5%] White, and 9443 [16.0%] other or unknown race) who delivered at 3 NewYork-Presbyterian hospitals: WCMC, LMH, and BMH. With the inclusion criteria described in the Methods section, we obtained a cohort with a total of 58 839 pregnancies, including 35 895 from WCMC, 8664 from LMH, and 14 280 from BMH. Across all sites, preeclampsia pregnancies were characterized by older maternal age (median [IQR] age, 35.0 [31.0–38.0] vs 34.0 [31.0-37.0] years in the WCMC group [*P* < .001], 35.0 [31.0–38.0] years vs 34.0 [32.0-37.0] years in the LMH group [*P* = .003], and 33.0 [28.0–36.0] vs 31.0 [26.0–35.0] years in the BMH group [*P* < .001]), greater proportions of Black race (335 of 2227 [15.0%] vs 2178 of 3668 [6.5%] in the WCMC group [*P* < .001], 117 of 792 [14.8%] vs 566 of 7872 [7.2%] in the LMH group [*P* < .001], and 455 of 1088 [41.8%] vs 2874 of 13,192 [21.8%] in the BMH group [*P* < .001]), nulliparity, multifetal gestation, and higher pregravid body mass index compared with controls (Table 1). Preeclampsia pregnancies were also associated with earlier delivery (median [IQR] delivery GA, 37.6 [36.6-39.1] vs 39.3 [38.6-40.0] weeks in the WCMC group [*P* < .001], 38.1 [37.1-39.4] vs 39.3 [38.6-39.9] weeks in the LMH group [*P* < .001], and 37.7 [36.7-39.3] vs 39.6 [38.7-40.4] weeks in the BMH group [*P* < .001]) and higher cesarean delivery rates (1231 of 2227 [55.3%] vs 10 853 of 33 668 [32.2%] in the WCMC group [*P* < .001], 328 of 792 [32.9%] vs 2275 of 7872 [28.9%] in the LMH group [*P* < .001], and 551 of 1088 [50.6%] vs 3229 of 13 192 [24.5%] in the BMH group [*P* < .001]). GA distribution of preeclampsia onset is shown in eFigure 3 in Supplement 1.

### Prediction Performance Across Gestational Windows

The rolling-window design revealed dynamic changes in prediction performance across gestation. Performance peaked at approximately 32 to 34 weeks, decreased at 38 weeks, and rebounded near delivery (Table 2; eTable 5 and eFigure 2 in Supplement 1). At the training site (WCMC; 2-week prediction window), the mean (SD) AUC increased from 0.803 (0.061) at 28 weeks to 0.863 (0.018) at 32 weeks and 0.866 (0.015) at 34 weeks before decreasing to 0.756 (0.014) at 38 weeks and recovering to 0.850 (0.020) at 40 weeks. Specificity at 90% sensitivity followed the same pattern: improving from 0.321 (0.133) at 28 weeks to 0.569 (0.127) at 34 weeks, decreasing to 0.406 (0.046) at 38 weeks, and increasing again to 0.586 (0.104) at 40 weeks. The PPV was low in early observation period (0.002 [0.000] at 28 weeks) but increased to 0.018 (0.002) at 34 weeks and peaked at 0.032 (0.002) at 38 weeks (approximately 2-fold to the corresponding prevalence). Across all time windows, the NPV remained consistently high (≥0.996). The results on the other evaluation metrics (ie, AUPRC and Brier score) are reported in eFigure 4 in Supplement 1. Additionally, when compared with a conventional risk score–based model constructed using commonly reported demographic and clinical risk factors, the model consistently achieved a higher PPV (approximately 2-fold) (eTable 6 in Supplement 1).

**Table 2.** Prediction Performance Across Gestational Windows, Prediction Windows, and Cross-Site Strategies^a^

Observation window	WCMC					LMH					BMH				
	AUC, mean (SD)	Specificity, mean (SD)	PPV, mean (SD)	NPV, mean (SD)	Prevalence	AUC, mean (SD)	Specificity, mean (SD)	PPV, mean (SD)	NPV, mean (SD)	Prevalance	AUC, mean (SD)	Specificity, mean (SD)	PPV, mean (SD)	NPV, mean (SD)	Prevalence
**1-wk Prediction window**															
0-28	0.807 (0.090)	0.524 (0.229)	0.002 (0.001)	1.000 (0.000)	0.0006	0.816 (0.090)	0.433 (0.050)	0.002 (0.000)	1.000 (0.000)	0.0005	0.684 (0.066)	0.384 (0.056)	0.001 (0.001	1.000 (0.000)	0.0006
0-32	0.855 (0.033)	0.675 (0.045)	0.005 (0.001)	1.000 (0.000)	0.0018	0.794 (0.014)	0.457 (0.073)	0.004 (0.004)	0.999 (0.001)	0.0022	0.776 (0.063)	0.399 (0.149)	0.004 (0.001)	0.999 (0.001)	0.0028
0-36	0.883 (0.017)	0.648 (0.082)	0.032 (0.007)	0.998 (0.000)	0.0120	0.838 (0.037)	0.483 (0.052)	0.028 (0.004)	0.996 (0.001)	0.0162	0.778 (0.029)	0.287 (0.064)	0.018 (0.003)	0.995 (0.001)	0.0143
0-40	0.807 (0.090)	0.578 (0.088)	0.015 (0.003)	0.999 (0.000)	0.0066	0.816 (0.090)	0.730 (0.000)	0.035 (0.011)	0.998 (0.001)	0.0107	0.684 (0.066)	0.453 (0.167)	0.013 (0.000)	0.998 (0.000)	0.0076
**2-wk Prediction window**															
0-28	0.803 (0.061)	0.321 (0.133)	0.002 (0.000)	0.999 (0.000)	0.0013	0.775 (0.099)	0.421 (0.033)	0.001 (0.002)	1.000 (0.000)	0.0007	0.749 (0.092)	0.302 (0.179)	0.002 (0.000)	1.000 (0.000)	0.0013
0-32	0.863 (0.018)	0.569 (0.127)	0.009 (0.003)	0.999 (0.000)	0.0041	0.833 (0.014)	0.424 (0.161)	0.007 (0.003)	0.998 (0.000)	0.0050	0.806 (0.016)	0.471 (0.000)	0.009 (0.000)	0.999 (0.001)	0.0052
0-36	0.856 (0.021)	0.557 (0.076)	0.047 (0.008)	0.996 (0.001)	0.0229	0.841 (0.016)	0.473 (0.000)	0.057 (0.012)	0.993 (0.002)	0.0325	0.798 (0.014)	0.455 (0.000)	0.046 (0.007)	0.993 (0.001)	0.0283
0-40	0.850 (0.020)	0.586 (0.104)	0.016 (0.004)	0.999 (0.000)	0.0070	0.890 (0.016)	0.740 (0.000)	0.040 (0.000)	0.998 (0.000)	0.0114	0.808 (0.029)	0.562 (0.122)	0.020 (0.007)	0.998 (0.001)	0.0093
**4-wk Prediction window**															
0-28	0.810 (0.043)	0.493 (0.111)	0.006 (0.001)	0.999 (0.000)	0.0034	0.783 (0.088)	0.384 (0.237)	0.006 (0.005)	0.999 (0.000)	0.0030	0.776 (0.044)	0.486 (0.000)	0.008 (0.000)	0.999 (0.000)	0.0045
0-32	0.860 (0.024)	0.557 (0.068)	0.026 (0.004)	0.998 (0.001)	0.0126	0.824 (0.014)	0.520 (0.000)	0.028 (0.007)	0.997 (0.000)	0.0142	0.803 (0.015)	0.428 (0.116)	0.024 (0.007)	0.996 (0.000)	0.0151
0-36	0.789 (0.016)	0.415 (0.035)	0.064 (0.004)	0.989 (0.001)	0.0422	0.755 (0.032)	0.385 (0.087)	0.097 (0.015)	0.997 (0.000)	0.0684	0.773 (0.019)	0.417 (0.034)	0.076 (0.005)	0.996 (0.001)	0.0507
0-40	0.845 (0.026)	0.580 (0.094)	0.016 (0.004)	0.999 (0.0)	0.0071	0.890 (0.016)	0.740 (0.000)	0.039 (0.010)	0.998 (0.001)	0.0114	0.808 (0.025)	0.562 (0.102)	0.021 (0.006)	0.998 (0.000)	0.0093

Abbreviations: BMH, Brooklyn Methodist Hospital; LMH, Lower Manhattan Hospital; WCMC, Weill Cornell Medical College.

^a^ Clinical performance metrics were calculated at a fixed sensitivity of 90%. This table presents a simplified summary of selected observation windows. The listed external validation results are from the model direct transfer to LMH and BMH, with the full results, including the retrained and rebuilt models, provided in eTable 5 in Supplement 1.

The external validation sites (LMH and BMH) exhibited similar trajectories. At LMH (model direct transfer, 2-week window), the mean (SD) AUC improved from 0.775 (0.099) at 28 weeks to peak performance of 0.834 (0.053) at 34 weeks, decreased to 0.716 (0.017) at 38 weeks, and rebounded strongly to 0.890 (0.016) at 40 weeks. Similarly, at BMH, the AUC improved from 0.749 (0.092) at 28 weeks to 0.806 (0.016) at 32 weeks and 0.808 (0.014) at 34 weeks, decreased to 0.729 (0.026) at 38 weeks, and rebounded to 0.808 (0.029) at 40 weeks. Specificity presented initial improvement from 28 to 34 weeks, followed by a decrease at 38 weeks, and recovery at 40 weeks. The PPV increased from approximately 0.001 to 0.002 at 28 weeks to peak values at 36 weeks (0.057 [0.012] at LMH and 0.046 [0.007] at BMH). The NPV remained high across all GAs (≥0.993).

Site-specific fine-tuning or retraining restored performance. At LMH (34-week observation, 2-week prediction window), model fine-tuning improved the mean (SD) AUC from 0.834 (0.053) to 0.868 (0.054) and the mean (SD) PPV from 0.016 (0.000) to 0.023 (0.000), whereas model retraining further boosted the AUC to 0.871 (0.017) and the PPV to 0.031 (0.020). The patterns were similar at BMH and LMH. The detailed results are reported in eTable 5 in Supplement 1.

In addition, the Cox proportional hazards regression model achieved discrimination performance comparable with the primary binary prediction framework across in-site and external validation settings. Detailed C-index results are shown in eFigure 11 in Supplement 1.

### Model Interpretability

**Figure 1.**
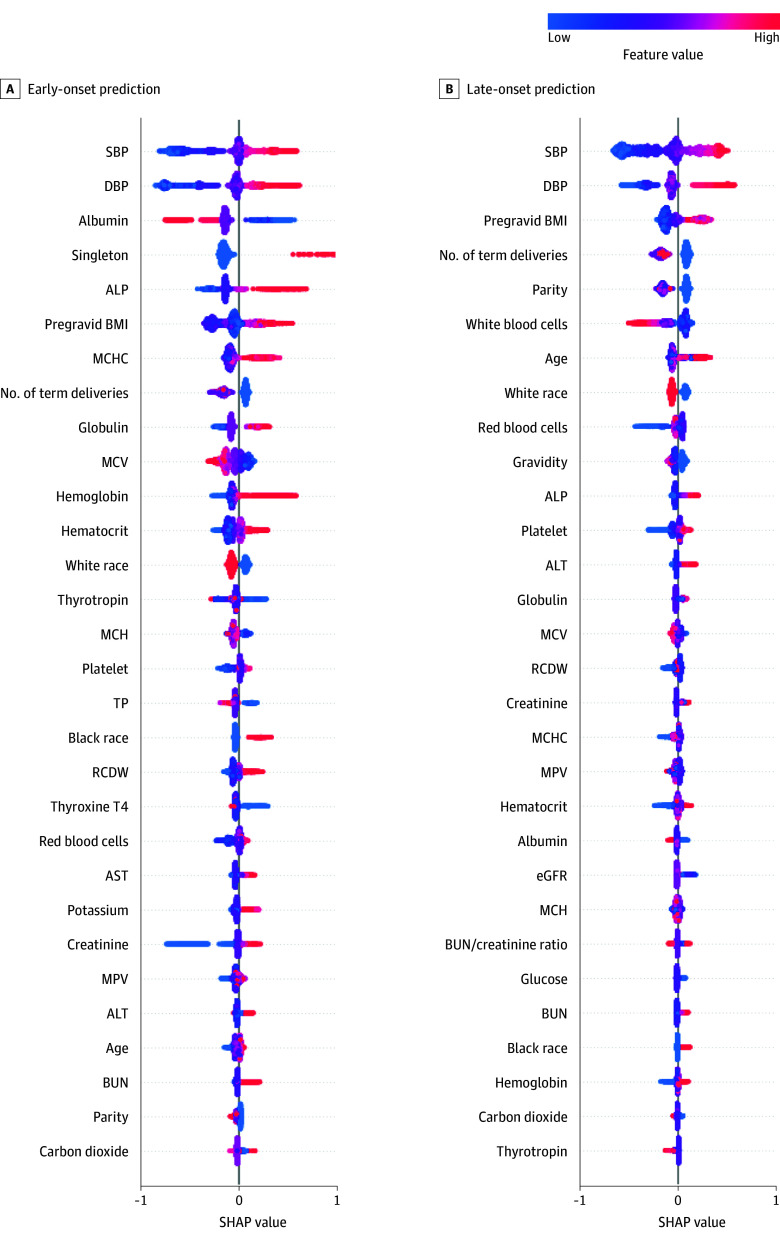
Top 30 Global Feature Importance for Preeclampsia Prediction at Early and Late Gestational Stages Global Shapley Additive Explanations (SHAP) analyses were performed on extreme gradient boosting models trained at Weill Cornell Medical College. A, Observation window up to 32 weeks with a 2-week prediction window (32-34 weeks), representing early-onset prediction. B, Observation window up to 38 weeks with a 2-week prediction window (38-40 weeks), representing late-onset prediction. For the SHAP summary plots, each dot represents an individual pregnancy and colors indicate feature values (with red indicating high and blue indicating low). The feature value plots display the top 30 features ranked by mean absolute SHAP value. Across both settings, systolic blood pressure (SBP) and diastolic blood pressure (DBP), pregravid body mass index (BMI), and obstetric history variables (parity and number of term deliveries) consistently ranked among the strongest predictors. Albumin and alkaline phosphatase (ALP) contributed more prominently at 32 weeks, whereas age, white blood cell count, and gravidity gained importance by 38 weeks, highlighting stage-specific difference. ALT indicates alanine aminotransferase; AST, aspartate aminotransferase; BUN, blood urea nitrogen; eGFR, estimated glomerular filtration rate; MCH, mean corpuscular hemoglobin; MCHC, mean corpuscular hemoglobin concentration; MCV, mean corpuscular volume; MPV, mean platelet volume; RCDW, red cell distribution width; TP, total protein.

BP was the dominant predictor across both early and late prediction windows.^24,25,26^ We observed that systolic and diastolic BP ranked first and second by mean SHAP values, remaining well above all other features (Figure 1 for WCMC; eFigures 5-10 in Supplement 1 for LMH and BMH).

At 32 to 34 weeks, laboratory features had greater influence compared with 38 to 40 weeks, supplementing BP as key contributors. Albumin was the third-ranked feature, followed by alkaline phosphatase and singleton pregnancy. Additional laboratory features such as globulin, mean corpuscular hemoglobin concentration, and mean corpuscular volume, as well as hematologic measures, including hemoglobin and hematocrit, all ranked within the top 15. These results indicate that in the early third trimester, biochemical and hematologic markers provided substantial predictive value alongside BP. At 38 to 40 weeks, demographic and obstetric factors were becoming more important, whereas the importance of laboratory markers decreased. Pregravid body mass index, number of term deliveries, and parity were all ranked in the top 5, surpassing many laboratory features. White blood cell count ranked 6th, maternal age ranked 7th, and gravidity ranked 10th. Meanwhile, albumin decreased from 3rd at 32 to 34 weeks to 21st at 38 to 40 weeks, whereas creatinine improved from 24th to 17th, and alanine transaminase increased from 26th to 13th. At 32 to 34 weeks, 5 of the top 10 features were laboratory or hematologic markers vs only 2 at 38 to 40 weeks.

### Sensitivity Analyses

**Figure 2.**
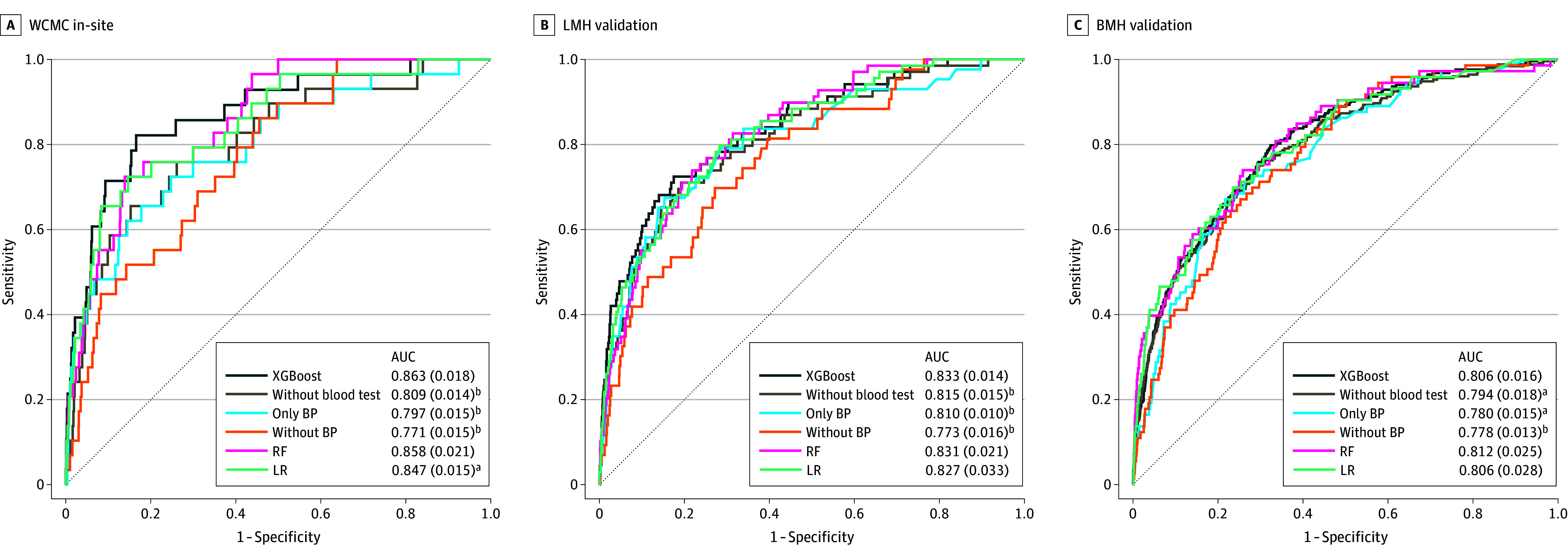
AUC Graphs Showing Results of Sensitivity Analyses of Model Variants Across Sites and Gestational Stages (0-32 Weeks) The figure shows results using an observation window up to 32 weeks of gestation and a 2-week prediction window (32-34 weeks). Receiver operating characteristic curves are shown for Weill Cornell Medical College (WCMC), Lower Manhattan Hospital (LMH), and Brooklyn Methodist Hospital (BMH). Default models (extreme gradient boosting [XGBoost]) were compared with models excluding blood test features (without blood test), only including blood pressure measurements (only BP), excluding blood pressure measurements (without BP), logistic regression, and random forest. Mean (SE) area under the receiver operating characteristic curve (AUC) is reported in each panel. Statistical significance of performance differences was evaluated against the default XGBoost model using the Welch *t* test. Across all sites, excluding blood test features or blood pressure measurements as well as only using blood pressure measurements significantly reduced AUC, whereas logistic regression (LR) and random forest (RF) yielded performance comparable to XGBoost in most comparisons. ^a^*P* < .05. ^b^*P* < .001.

**Figure 3.**
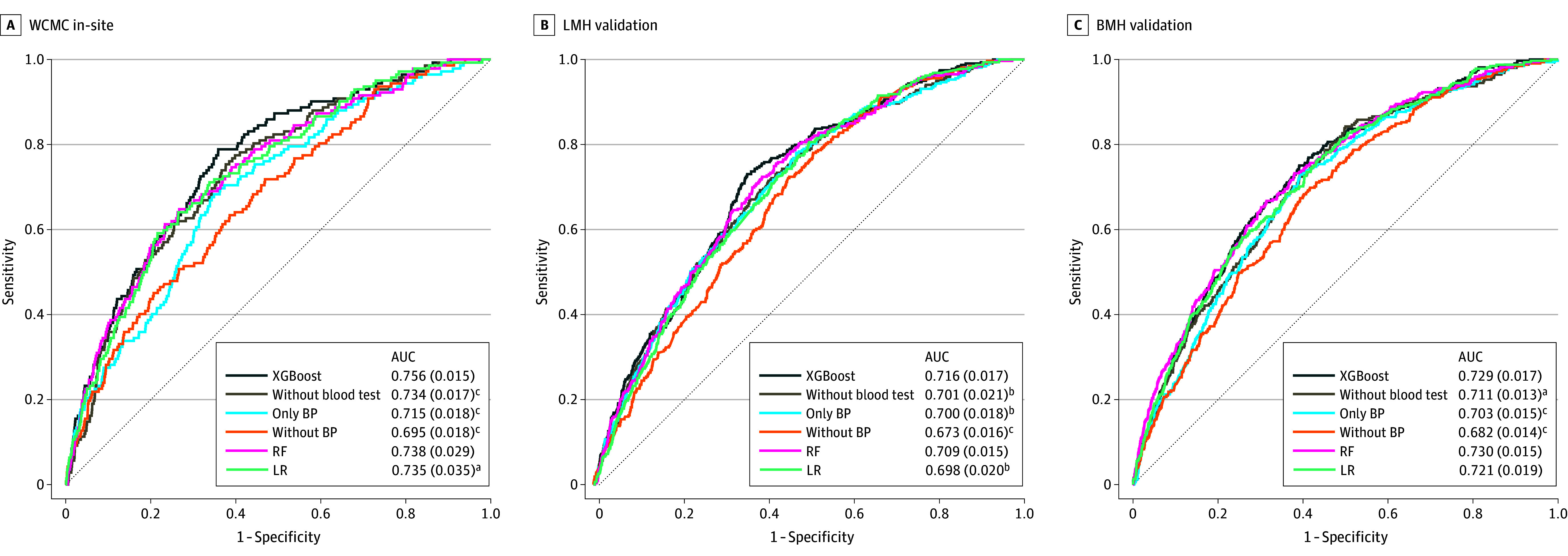
AUC Graphs Showing Results of Sensitivity Analyses of Model Variants Across Sites and Gestational Stages (0-38 Weeks) The figure shows results using an observation window up to 38 weeks of gestation and a 2-week prediction window (38-40 weeks). Receiver operating characteristic curves are shown for Weill Cornell Medical College (WCMC), Lower Manhattan Hospital (LMH), and Brooklyn Methodist Hospital (BMH). Default models (extreme gradient boosting [XGBoost]) were compared with models excluding blood test features (without blood test), only including blood pressure measurements (only BP), excluding blood pressure measurements (without BP), logistic regression (LR), and random forest (RF). Mean (SE) area under the receiver operating characteristic curve (AUC) is reported in each panel. Statistical significance of performance differences was evaluated against the default XGBoost model using the Welch *t* test. Across all sites, excluding blood test features or blood pressure measurements as well as only using blood pressure measurements significantly reduced AUC, whereas logistic regression (LR) and random forest (RF) yielded performance comparable to XGBoost in most comparisons. ^a^*P* < .05. ^b^*P* < .01. ^c^*P* < .001.

As shown in Figure 2 and Figure 3, excluding blood test features, excluding BP features, or restricting the models to BP only resulted in performance decreases. At 32 weeks’ observation with a 2-week prediction window, the BP-only model achieved mean (SD) AUCs of 0.797 (0.015), 0.810 (0.010), and 0.780 (0.015) at WCMC, LMH and BMH, respectively, whereas the full model achieved higher AUCs of 0.863 (0.018), 0.833 (0.014), and 0.806 (0.016), respectively.

Logistic regression, random forest and deep learning–based models did not perform better than our XGBoost model, indicating that the prediction performance is not sensitive to the model choice. The patient-level evaluation and feature exclusion based on missingness also did not result in consistent performance gains (eTable 3 in Supplement 1). Stratification analysis on key demographic subgroups (eg, age and race) shows no substantial fairness concerns (eTable 4 in Supplement 1).

## Discussion

Preeclampsia remains a critical obstetric challenge due to its unpredictable onset, rapid progression, and disproportionate burden in low-resource settings.^27,28^ Most prior efforts have focused on first-trimester risk stratification to guide low-dose aspirin prophylaxis and enhanced monitoring.^29,30^ Although aspirin prophylaxis can reduce the incidence of preterm preeclampsia when initiated before 16 weeks at appropriate doses, its effect on late-onset and term preeclampsia, which constitute most cases, has been minimal.^31,32,33^ As a result, current prediction strategies face a mismatch between clinical need and predictive capability. First-trimester prediction models achieve high performance for early-onset preeclampsia (AUC >0.8) but only modest accuracy for late-onset and term preeclampsia (AUC of approximately 0.6-0.7). Our study directly addresses these gaps by shifting the focus to late pregnancy, when most cases arise. By implementing rolling observation and prediction windows, our models not only identify who is at risk but also estimate when preeclampsia is likely to occur within 1 to 4 weeks. This ability to capture both occurrence and timing provides clinically actionable lead time, aligning prediction with the timing of routine prenatal care and supporting timely intervention.

Currently, there is no feasible way to efficiently perform a longitudinal risk assessment continually as the pregnancy progresses. A dynamic rolling-window approach addresses this need by continually updating the risk prediction using recent data rather than treating risk as a fixed probability determined months in advance. Our rolling window design differs from existing studies, which typically treat preeclampsia risk as static, providing single estimates at fixed time points or across broad gestational stages.^8,34^ Previous EHR-based studies^19,35,36^ have started to model pregnancy trajectories; they operate at coarse temporal resolution and focus on overall pregnancy outcomes rather than short-term risk estimation. Our framework generates risk estimation at a much finer temporal resolution in terms of GAs throughout late pregnancy, revealing a previously unrevealed pattern in predictive performance. We observed a consistent increase-peak-decrease performance pattern across all sites, where AUC and specificity (at 90% sensitivity) improved from 28 to 34 weeks, decreased at 38 weeks, and then rebounded near delivery. This pattern aligns with prior studies reporting that prediction of the predominant term preeclampsia remains challenging, with accuracy decreasing as the GA at onset advances.^37,38^ Importantly, this rolling-window design naturally stratifies patients by early-onset, preterm, and term preeclampsia without requiring predefined clinical labels, enhancing clinical applicability. Routine prenatal visits, which happen with more frequency as the pregnancy advances, generate a wealth of longitudinal data that can be used as part of a constantly updating prediction model to give clinicians an updated estimated risk of the patient developing preeclampsia in the near future. This is clinically useful because it aligns with decision-making time frames: obstetric specialists typically make week-by-week or visit-by-visit management decisions in late pregnancy.

PPVs were modest, reflecting the low incidence of preeclampsia. Importantly, PPVs of our models demonstrated 2- to 4-fold increases compared with the corresponding preeclampsia prevalence across all GAs. PPVs also increased consistently with advancing GA and peaked near delivery, when clinical decision-making is most critical. Even small absolute gains in PPV at this stage may translate into meaningful reductions in unnecessary interventions while preserving high NPVs. Additionally, the model consistently achieved higher PPV (approximately 2-fold) compared with a risk score–based model, suggesting that incorporating longitudinal physiologic and laboratory data substantially improves the clinical yield of short-term preeclampsia prediction beyond static risk factor–based approaches.

The predictors included maternal characteristics, BP, and routine laboratory panels that are widely available in routine EHRs, supporting the practicality of the model in diverse clinical settings and GAs. Across sites, BP emerged as the dominant predictor of preeclampsia, consistent with its central role in disease pathophysiology. Adding routine laboratory features further enhanced model performance across sites and gestational windows. At 32 to 34 weeks, inclusion of laboratory tests improved AUCs by approximately 0.05 compared with models using BP and other maternal characteristics alone, and similar gains were observed at later windows. Feature rankings also shifted with GA. At approximately 32 weeks, protein-related and hematologic markers (albumin, globulin, alkaline phosphatase, and mean corpuscular hemoglobin concentration) were among the strongest contributors, consistent with prior reports^39,40,41^ linking early-onset preeclampsia to endothelial dysfunction and placental insufficiency. By 38 weeks, obstetric history, maternal age, and indicators of inflammation and hepatic stress (eg, white blood cells and alanine transaminase) became more influential, aligning with the notion that late-onset preeclampsia is more strongly driven by maternal metabolic and inflammatory pathways.^41,42^ Although the precise mechanisms remain incompletely understood, these shifts suggest that routine laboratory features not only enhance model performance but also may reflect biologically distinct processes across the spectrum of preeclampsia.

We have also tested the generalizability of our model. When the WCMC-trained model was directly applied to LMH and BMH, it maintained good discrimination performance (AUC >0.7 as defined in existing work), demonstrating the cross-site transferability of our model. We further evaluated local adaptation strategies to determine whether they could enhance performance beyond direct transfer. Both strategies, including model fine-tuning and model retraining, provided performance gains, indicating that although the model generalizes well across sites, local adaptation can further optimize performance when site-specific data are available. In addition, the generalizability of our model was further demonstrated by the demographic diversity across hospitals. Each hospital served a distinct patient population. WCMC was mostly White, LMH had a larger proportion of Asian patients, and BMH included the highest proportion of Black patients. Preeclampsia prevalence and severity are known to differ across racial groups.^17,43,44^ Black women have consistently shown higher odds of preeclampsia and severity compared with White women,^45^ whereas Asian women often have lower relative risk.^17^ Applying the model across such demographic contrasts allowed us to evaluate its performance under true heterogeneity in both incidence and outcomes. Despite these differences, performance remained stable across all hospitals, demonstrating the generalizability of our model across diverse demographic groups.

### Limitations 

This study has several limitations. First, it was retrospective and conducted within a single health care system, although across 3 demographically distinct hospitals. Validation in more diverse patient populations, particularly in low- and middle-income countries, will be essential. Second, although we analyzed more than 58 000 pregnancies, the longitudinal data had missing values and irregular sampling. Although this reflects clinical practice, the large sample size helps mitigate these gaps, and our models were designed to rely on either median BP or the most recent laboratory results together with static demographic and obstetric variables. Third, preeclampsia diagnoses were based on EHR documentation and *ICD-10* codes, which may introduce misdiagnosis, although the risk is partly offset by the large cohort size and the consistency across sites.

## Conclusions

This cohort study found that dynamic, short-term prediction of preeclampsia in late gestation is feasible using only routinely available clinical features. By providing advanced actionable time and relying on a simple, uniform feature set, our framework offers a pragmatic foundation for scalable implementation. These strengths highlight its potential to support more timely and accessible obstetric care across varied health care settings.

## Notes

Supplement 1. **eTable 1.** Summary of features used for model development **eTable 2.** Missingness summary of features used for model development **eTable 3.** Sensitivity analyses of model performance under alternative settings **eTable 4.** Subgroup performance of the prediction model by age and race **eTable 5.** Prediction performance across gestational windows, prediction windows, and cross-site strategies **eTable 6.** Comparison of machine learning–based models and a risk score–based model for prediction of preeclampsia **eFigure 1.** Study design and machine learning framework for preeclampsia (PE) prediction **eFigure 2.** Prediction performance across gestational windows, prediction windows, and cross-site strategies **eFigure 3.** The distribution of PE patient counts across gestational weeks **eFigure 4.** Prediction performance across gestational windows and cross-site strategies **eFigure 5.** Top-30 global feature importance for PE prediction at early and late gestational stages on LMH with direct transfer strategy **eFigure 6.** Top-30 global feature importance for PE prediction at early and late gestational stages on LMH with fine-tuning strategy **eFigure 7.** Top-30 global feature importance for PE prediction at early and late gestational stages on LMH with retraining strategy **eFigure 8.** Top-30 global feature importance for PE prediction at early and late gestational stages on BMH with direct transfer strategy **eFigure 9.** Top-30 global feature importance for PE prediction at early and late gestational stages on BMH with fine-tuning strategy **eFigure 10.** Top-30 global feature importance for PE prediction at early and late gestational stages on BMH with retraining strategy **eFigure 11.** Comparison of discrimination performance between the primary binary prediction framework and a Cox proportional hazards model across different settings
eMethods.

Supplement 2.
Data Sharing Statement
