# Supplementary material for: Machine Learning for Dynamic and Short-Term Prediction of Preeclampsia Using Routine Clinical Data
Source: JAMA Netw Open. 2026 Mar 6;9(3):e260359. doi: 10.1001/jamanetworkopen.2026.0359 (PMC12966928; doi:10.1001/jamanetworkopen.2026.0359)
Supplement: Supplement 1. — eTable 1. Summary of features used for model development eTable 2. Missingness summary of features used for model development eTable 3. Sensitivity analyses of model performance under alternative settings eTable 4. Subgroup performance of the prediction model by age and race eTable 5. Prediction performance across gestational windows, prediction windows, and cross-site strategies eTable 6. Comparison of machine learning–based models and a risk score–based model for prediction of preeclampsia eFigure 1. Study design and machine learning framework for preeclampsia (PE) prediction eFigure 2. Prediction performance across gestational windows, prediction windows, and cross-site strategies eFigure 3. The distribution of PE patient counts across gestational weeks eFigure 4. Prediction performance across gestational windows and cross-site strategies eFigure 5. Top-30 global feature importance for PE prediction at early and late gestational stages on LMH with direct transfer strategy eFigure 6. Top-30 global feature importance for PE prediction at early and late gestational stages on LMH with fine-tuning strategy eFigure 7. Top-30 global feature importance for PE prediction at early and late gestational stages on LMH with retraining strategy eFigure 8. Top-30 global feature importance for PE prediction at early and late gestational stages on BMH with direct transfer strategy eFigure 9. Top-30 global feature importance for PE prediction at early and late gestational stages on BMH with fine-tuning strategy eFigure 10. Top-30 global feature importance for PE prediction at early and late gestational stages on BMH with retraining strategy eFigure 11. Comparison of discrimination performance between the primary binary prediction framework and a Cox proportional hazards model across different settings eMethods. [file jamanetwopen-e260359-s001.pdf]

Supplemental Online Content

Li H, Li Y, Zang C, et al. Machine learning for dynamic and short-term prediction of preeclampsia using routine clinical data. *JAMA Netw Open*. 2026;9(3):e260359.
doi:10.1001/jamanetworkopen.2026.0359

eTable 1. Summary of features used for model development
eTable 2. Missingness summary of features used for model development
eTable 3. Sensitivity analyses of model performance under alternative settings
eTable 4. Subgroup performance of the prediction model by age and race
eTable 5. Prediction performance across gestational windows, prediction windows, and cross-site strategies
eTable 6. Comparison of machine learning–based models and a risk score–based model for prediction of preeclampsia
eFigure 1. Study design and machine learning framework for preeclampsia (PE) prediction
eFigure 2. Prediction performance across gestational windows, prediction windows, and cross-site strategies
eFigure 3. The distribution of PE patient counts across gestational weeks
eFigure 4. Prediction performance across gestational windows and cross-site strategies
eFigure 5. Top-30 global feature importance for PE prediction at early and late gestational stages on LMH with direct transfer strategy
eFigure 6. Top-30 global feature importance for PE prediction at early and late gestational stages on LMH with fine-tuning strategy
eFigure 7. Top-30 global feature importance for PE prediction at early and late gestational stages on LMH with retraining strategy
eFigure 8. Top-30 global feature importance for PE prediction at early and late gestational stages on BMH with direct transfer strategy
eFigure 9. Top-30 global feature importance for PE prediction at early and late gestational stages on BMH with fine-tuning strategy
eFigure 10. Top-30 global feature importance for PE prediction at early and late gestational stages on BMH with retraining strategy
eFigure 11. Comparison of discrimination performance between the primary binary prediction framework and a Cox proportional hazards model across different settings
eMethods. A full detailed Method section listed here

This supplemental material has been provided by the authors to give readers additional information about their work.

Supplementary Table

Supplementary eTable 1. Summary of features used for model development. Age, Race and lifestyle features are self-reported; Laboratory tests: Complete blood count panel was measured with Sysmex XN-9100; Comprehensive metabolic and hepatic function panels were measured with Siemens XPT; Thyroid function panel was measured with Siemens Centaur platform.

Category	Sub-Category	Features
Clinical characteristics	Demographics	Age; Race
	Obstetric factors	Gravidity; Parity; Number of term deliveries; Pregravid BMI; Singleton pregnancy
	Lifestyle	Tobacco use; Alcohol use
	Blood pressure	Systolic blood pressure (SBP); Diastolic blood pressure (DBP)
Laboratory tests (contains calculated results)	Complete blood count panel	WBC; RBC; Hemoglobin; Hematocrit; Platelet count; MCV; MCH; MCHC; RDW; MPV; NRBC
	Comprehensive metabolic and hepatic function panels	Calcium; Sodium; Potassium; Chloride; Bicarbonate; Anion gap; Glucose; Creatinine; BUN; BUN/Creatinine ratio; eGFR; Albumin, Total protein; ALT; AST; ALP; Total bilirubin; Direct bilirubin; Indirect bilirubin; Globulin; Albumin/Globulin ratio
	Thyroid function	TSH; Free T4

© 2026 Li H et al. *JAMA Network Open*.

Supplementary eTable 2. Missingness summary of features used for model development.

Category	Sub-Category	Features	Number of missing instances (%)		
			WCMC (N=35895)	LMH (N=8664)	BMH (N=14280)
Clinical characteristics	Demographics	Age	0 (0.00%)	0 (0.00%)	0 (0.00%)
		Race	2627 (7.32%)	594 (6.86%)	813 (5.69%)
	Obstetric factors	Gravidity; Parity; Number of term deliveries; Pregravid BMI; Singleton pregnancy	0 (0.00%)	0 (0.00%)	0 (0.00%)
	Lifestyle	Tobacco use; Alcohol use			
	Blood pressure	Systolic blood pressure (SBP); Diastolic blood pressure (DBP)	611 (1.70%)	369 (4.26%)	1640 (11.48%)
Laboratory tests (contains calculated results)	Complete blood Count panel	WBC; RBC	2445 (6.81%)	854 (9.86%)	2865 (20.07%)
		Hemoglobin; Hematocrit; MCV; MCH; MCHC; RDW	638 (1.78%)	442 (5.11%)	1695 (11.87%)
		Platelet count	2465 (6.87%)	859 (9.92%)	2887 (20.22%)
		MPV	733 (2.04%)	456 (5.26%)	1782 (12.48%)
		NRBC	2517 (7.01%)	3613 (41.70%)	13275 (92.96%)
	Comprehensive metabolic and Hepatic function panels	Calcium	20520 (57.17%)	5539 (63.93%)	8569 (60.00%)
		Sodium; Chloride; Creatinine	20508 (57.13%)	5534 (63.88%)	8563 (59.96%)
		Bicarbonate	20533 (57.20%)	5543 (63.97%)	8564 (59.97%)
		Potassium	20633 (57.48%)	5543 (63.97%)	8803 (61.65%)
		Anion gap	21914 (61.05%)	5923 (68.37%)	9064 (63.48%)
		Glucose	21974 (61.22%)	5698 (65.77%)	8580 (60.08%)
		BUN	20534 (57.21%)	5543 (63.97%)	8564 (59.97%)
		BUN/Creatinine ratio	21568 (60.09%)	5809 (67.05%)	11096 (77.71%)
		eGFR	32534 (90.64%)	7952 (91.78%)	13326 (93.32%)
		Albumin	20629 (57.47%)	5570 (64.29%)	10858 (76.03%)
		Total protein	20632 (57.48%)	5570 (64.29%)	10858 (76.03%)
		ALT	20665 (57.57%)	5579 (64.39%)	10862 (76.06%)
		AST	20764 (57.85%)	5580 (64.40%)	10861 (76.06%)
		ALP	20,629 (57.47%)	5570 (64.29%)	10859 (76.04%)
		Total bilirubin	20630 (54.47%)	5570 (64.29%)	10859 (76.04%)
		Globulin	20662 (57.56%)	5579 (64.39%)	10861 (76.06%)
		Albumin/Globulin ratio	32164 (89.61%)	7830 (90.37%)	13713 (96.03%)
		Direct bilirubin	31356 (87.35%)	6483 (74.82%)	11855 (83.02%)
		Indirect bilirubin	32827 (91.45%)	7179 (82.86%)	13837 (96.89%)
	Thyroid function	TSH	20170 (56.19%)	5510 (63.60%)	13008 (91.09%)
		Free T4	23712 (66.06%)	7094 (81.88%)	13876 (97.17%)

© 2026 Li H et al. *JAMA Network Open*.

Supplementary eTable 3. Sensitivity analyses of model performance under alternative settings. This table reports the mean area under the receiver operating characteristic curve (AUC) with standard error across two observation windows (up to 32 and 38 gestational weeks), corresponding 2-week prediction windows, and three evaluation sites (WCM, LMH, and BMH). Sensitivity analyses were conducted to assess the robustness of the primary pregnancy-level XGBoost model by varying (i) the modeling approach, including several deep learning–based tabular models; (ii) the unit of analysis, comparing pregnancy-level and patient-level evaluation; and (iii) the feature set through exclusion of variables exceeding predefined missingness thresholds.

**Mean AUC ± standard error**	Observation window up to 32 weeks and a 2-week prediction window (32–34 weeks)			Observation window up to 38 weeks and a 2-week prediction window (38–40 weeks)		
	WCMC	LMH	BMH	WCMC	LMH	BMH
**Primary analysis (XGBoost)**	0.863 ± 0.018	0.833 ± 0.014	0.806 ± 0.016	0.756 ± 0.015	0.716 ± 0.017	0.729 ± 0.017
Deep learning models						
**TabPFN**[1]	0.857 ± 0.022	0.830 ± 0.023	0.795 ± 0.020	0.757 ± 0.019	0.711 ± 0.026	0.728 ± 0.022
**TabM**[2]	0.856 ± 0.016	0.833 ± 0.019	0.791 ± 0.029	0.753 ± 0.034	0.707 ± 0.025	0.723 ± 0.030
**TabTransformer**[3]	0.865 ± 0.017	0.835 ± 0.020	0.802 ± 0.024	0.756 ± 0.024	0.692 ± 0.030	0.729 ± 0.019
**SAINT**[4]	0.852 ± 0.015	0.831 ± 0.019	0.798 ± 0.021	0.752 ± 0.014	0.703 ± 0.019	0.727 ± 0.020
**AutoInt**[5]	0.858 ± 0.031	0.825 ± 0.025	0.791 ± 0.027	0.751 ± 0.023	0.708 ± 0.026	0.723 ± 0.029
The unit of analysis						
**Patient-level analysis**	0.860 ± 0.019	0.834 ± 0.017	0.802 ± 0.021	0.757 ± 0.012	0.721 ± 0.021	0.725 ± 0.019
Feature filtering by missingness threshold						
**Excluding features with missingness > 0.9**	0.863 ± 0.017	0.832 ± 0.015	0.806 ± 0.017	0.757 ± 0.013	0.714 ± 0.017	0.728 ± 0.015
**Excluding features with missingness > 0.7**	0.856 ± 0.022	0.823 ± 0.026	0.802 ± 0.028	0.751 ± 0.021	0.708 ± 0.017	0.720 ± 0.017
**Excluding features with missingness > 0.5**	0.851 ± 0.018	0.821 ± 0.021	0.795 ± 0.023	0.742 ± 0.016	0.701 ± 0.015	0.712 ± 0.021

© 2026 Li H et al. *JAMA Network Open*.

Supplementary eTable 4. Subgroup performance of the prediction model by age and race. This table reports the mean area under the receiver operating characteristic curve (AUC) with standard error for the primary XGBoost model, stratified by maternal age (<35 vs ≥35 years) and race (White, Black or African American, Asian). Results are shown for two observation windows (up to 32 and 38 gestational weeks) with corresponding 2-week prediction windows, evaluated on the in-site cohort (WCMC) and two external validation cohorts (LMH and BMH). Comparable discrimination performance across age and race subgroups on external validation datasets suggests consistent model performance across population subgroups.

**Mean AUC ± standard error**	**Observation window up to 32 weeks and a 2-week prediction window (32–34 weeks)**			**Observation window up to 38 weeks and a 2-week prediction window (38–40 weeks)**		
	WCMC	LMH	BMH	WCMC	LMH	BMH
**Primary analysis**	0.863 ± 0.018	0.833 ± 0.014	0.806 ± 0.016	0.756 ± 0.015	0.716 ± 0.017	0.729 ± 0.017
Age						
**Age < 35**	0.857 ± 0.017	0.828 ± 0.012	0.799 ± 0.017	0.748 ± 0.018	0.709 ± 0.018	0.720 ± 0.012
**Age ≥ 35**	0.869 ± 0.020	0.839 ± 0.021	0.810 ± 0.014	0.762 ± 0.013	0.725 ± 0.015	0.736 ± 0.019
Race						
**White**	0.864 ± 0.015	0.831 ± 0.016	0.803 ± 0.010	0.752 ± 0.018	0.718 ± 0.012	0.725 ± 0.019
**Black or African American**	0.853 ± 0.014	0.820 ± 0.013	0.798 ± 0.016	0.751 ± 0.021	0.702 ± 0.011	0.718 ± 0.015
**Asian**	0.854 ± 0.017	0.823 ± 0.018	0.801 ± 0.013	0.753 ± 0.031	0.716 ± 0.013	0.722 ± 0.017

© 2026 Li H et al. *JAMA Network Open*.

Supplementary eTable 5. Prediction performance across gestational windows, prediction windows, and cross-site strategies.

Week	Delta	Metric	WCMC	LMH (direct transfer)	LMH (fine-tuning)	LMH (retraining)	BMH (direct transfer)	BMH (fine-tuning)	BMH (retraining)
28	1	AUC	0.807 (0.090)	0.816 (0.090)	0.820 (0.091)	0.825 (0.090)	0.684 (0.066)	0.694 (0.063)	0.700 (0.014)
30	1	AUC	0.800 (0.022)	0.847 (0.043)	0.885 (0.042)	0.894 (0.022)	0.709 (0.032)	0.716 (0.032)	0.744 (0.017)
32	1	AUC	0.855 (0.033)	0.794 (0.014)	0.792 (0.016)	0.806 (0.046)	0.776 (0.063)	0.786 (0.065)	0.810 (0.059)
34	1	AUC	0.859 (0.038)	0.809 (0.020)	0.829 (0.021)	0.844 (0.015)	0.787 (0.046)	0.837 (0.044)	0.840 (0.015)
36	1	AUC	0.883 (0.017)	0.838 (0.037)	0.855 (0.037)	0.866 (0.016)	0.778 (0.029)	0.817 (0.028)	0.820 (0.038)
38	1	AUC	0.755 (0.021)	0.695 (0.021)	0.706 (0.022)	0.712 (0.014)	0.729 (0.054)	0.744 (0.052)	0.756 (0.030)
40	1	AUC	0.840 (0.036)	0.890 (0.017)	0.893 (0.016)	0.900 (0.020)	0.790 (0.017)	0.820 (0.017)	0.821 (0.025)
28	1	PPV @ 90% Sensitivity	0.002 (0.001)	0.002 (0.000)	0.002 (0.000)	0.003 (0.000)	0.001 (0.001)	0.001 (0.001)	0.001 (0.000)
30	1	PPV @ 90% Sensitivity	0.002 (0.000)	0.002 (0.009)	0.009 (0.009)	0.013 (0.004)	0.002 (0.001)	0.003 (0.001)	0.004 (0.003)
32	1	PPV @ 90% Sensitivity	0.005 (0.001)	0.004 (0.004)	0.011 (0.004)	0.014 (0.006)	0.004 (0.001)	0.004 (0.001)	0.005 (0.001)
34	1	PPV @ 90% Sensitivity	0.010 (0.005)	0.005 (0.014)	0.012 (0.013)	0.015 (0.006)	0.008 (0.000)	0.009 (0.000)	0.018 (0.017)
36	1	PPV @ 90% Sensitivity	0.032 (0.007)	0.028 (0.004)	0.030 (0.004)	0.034 (0.007)	0.018 (0.003)	0.024 (0.003)	0.027 (0.006)
38	1	PPV @ 90% Sensitivity	0.017 (0.001)	0.029 (0.004)	0.030 (0.004)	0.031 (0.004)	0.018 (0.000)	0.020 (0.000)	0.021 (0.004)
40	1	PPV @ 90% Sensitivity	0.015 (0.003)	0.035 (0.011)	0.040 (0.011)	0.040 (0.015)	0.013 (0.000)	0.016 (0.000)	0.016 (0.007)
28	1	Specificity @ 90% Sensitivity	0.524 (0.229)	0.433 (0.050)	0.518 (0.052)	0.535 (0.047)	0.384 (0.056)	0.389 (0.058)	0.431 (0.072)
30	1	Specificity @ 90% Sensitivity	0.572 (0.079)	0.435 (0.061)	0.531 (0.062)	0.558 (0.057)	0.389 (0.209)	0.390 (0.208)	0.433 (0.075)
32	1	Specificity @ 90% Sensitivity	0.675 (0.045)	0.457 (0.073)	0.548 (0.074)	0.609 (0.070)	0.399 (0.149)	0.419 (0.152)	0.434 (0.078)
34	1	Specificity @ 90% Sensitivity	0.592 (0.129)	0.420 (0.000)	0.421 (0.000)	0.529 (0.241)	0.361 (0.000)	0.480 (0.000)	0.563 (0.192)
36	1	Specificity @ 90% Sensitivity	0.648 (0.082)	0.483 (0.052)	0.516 (0.053)	0.562 (0.098)	0.287 (0.064)	0.464 (0.065)	0.492 (0.138)
38	1	Specificity @ 90% Sensitivity	0.377 (0.037)	0.331 (0.091)	0.352 (0.091)	0.354 (0.083)	0.327 (0.155)	0.376 (0.151)	0.391 (0.000)
40	1	Specificity @ 90% Sensitivity	0.578 (0.088)	0.730 (0.000)	0.731 (0.000)	0.745 (0.074)	0.453 (0.167)	0.496 (0.167)	0.570 (0.000)
28	2	AUC	0.803 (0.061)	0.775 (0.099)	0.781 (0.098)	0.795 (0.086)	0.749 (0.092)	0.757 (0.088)	0.762 (0.079)
30	2	AUC	0.829 (0.015)	0.791 (0.070)	0.808 (0.073)	0.812 (0.063)	0.806 (0.015)	0.812 (0.014)	0.812 (0.065)
32	2	AUC	0.863 (0.018)	0.833 (0.014)	0.849 (0.014)	0.849 (0.023)	0.806 (0.016)	0.828 (0.015)	0.834 (0.072)
34	2	AUC	0.866 (0.015)	0.834 (0.053)	0.868 (0.054)	0.871 (0.017)	0.808 (0.014)	0.811 (0.015)	0.820 (0.031)
36	2	AUC	0.856 (0.021)	0.841 (0.016)	0.847 (0.014)	0.848 (0.015)	0.798 (0.014)	0.800 (0.015)	0.817 (0.029)
38	2	AUC	0.756 (0.015)	0.716 (0.017)	0.717 (0.017)	0.721 (0.017)	0.729 (0.017)	0.750 (0.026)	0.754 (0.044)
40	2	AUC	0.850 (0.020)	0.890 (0.016)	0.899 (0.017)	0.907 (0.017)	0.808 (0.029)	0.826 (0.028)	0.827 (0.014)
28	2	PPV @ 90% Sensitivity	0.002 (0.000)	0.001 (0.002)	0.003 (0.002)	0.008 (0.006)	0.002 (0.000)	0.002 (0.000)	0.002 (0.000)
30	2	PPV @ 90% Sensitivity	0.004 (0.001)	0.004 (0.003)	0.005 (0.003)	0.006 (0.005)	0.005 (0.000)	0.006 (0.000)	0.006 (0.002)
32	2	PPV @ 90% Sensitivity	0.009 (0.003)	0.007 (0.003)	0.009 (0.003)	0.011 (0.000)	0.009 (0.000)	0.010 (0.000)	0.014 (0.008)
34	2	PPV @ 90% Sensitivity	0.018 (0.002)	0.016 (0.000)	0.023 (0.000)	0.031 (0.020)	0.017 (0.003)	0.017 (0.003)	0.018 (0.005)
36	2	PPV @ 90% Sensitivity	0.047 (0.008)	0.057 (0.012)	0.058 (0.012)	0.062 (0.011)	0.046 (0.007)	0.047 (0.007)	0.048 (0.007)

© 2026 Li H et al. *JAMA Network Open*.

38	2	PPV @ 90% Sensitivity	0.032 (0.002)	0.053 (0.004)	0.055 (0.004)	0.062 (0.005)	0.032 (0.003)	0.037 (0.003)	0.038 (0.007)
40	2	PPV @ 90% Sensitivity	0.016 (0.004)	0.040 (0.000)	0.043 (0.000)	0.047 (0.012)	0.020 (0.007)	0.022 (0.007)	0.022 (0.008)
28	2	Specificity @ 90% Sensitivity	0.321 (0.133)	0.421 (0.033)	0.475 (0.034)	0.496 (0.027)	0.302 (0.179)	0.371 (0.171)	0.376 (0.000)
30	2	Specificity @ 90% Sensitivity	0.435 (0.128)	0.407 (0.231)	0.491 (0.225)	0.501 (0.000)	0.370 (0.147)	0.494 (0.152)	0.511 (0.000)
32	2	Specificity @ 90% Sensitivity	0.569 (0.127)	0.424 (0.161)	0.426 (0.169)	0.614 (0.000)	0.471 (0.000)	0.549 (0.000)	0.560 (0.165)
34	2	Specificity @ 90% Sensitivity	0.562 (0.057)	0.444 (0.230)	0.589 (0.225)	0.627 (0.000)	0.438 (0.000)	0.448 (0.000)	0.456 (0.128)
36	2	Specificity @ 90% Sensitivity	0.557 (0.076)	0.473 (0.000)	0.501 (0.000)	0.518 (0.106)	0.455 (0.000)	0.459 (0.000)	0.470 (0.086)
38	2	Specificity @ 90% Sensitivity	0.406 (0.046)	0.327 (0.054)	0.355 (0.055)	0.427 (0.052)	0.316 (0.124)	0.401 (0.129)	0.406 (0.050)
40	2	Specificity @ 90% Sensitivity	0.586 (0.104)	0.740 (0.000)	0.768 (0.000)	0.779 (0.053)	0.562 (0.122)	0.580 (0.121)	0.588 (0.000)
28	4	AUC	0.810 (0.043)	0.783 (0.088)	0.833 (0.085)	0.857 (0.015)	0.776 (0.044)	0.796 (0.044)	0.800 (0.016)
30	4	AUC	0.848 (0.017)	0.797 (0.059)	0.839 (0.057)	0.842 (0.016)	0.801 (0.035)	0.831 (0.034)	0.836 (0.016)
32	4	AUC	0.860 (0.024)	0.824 (0.014)	0.851 (0.017)	0.854 (0.032)	0.803 (0.015)	0.812 (0.014)	0.819 (0.041)
34	4	AUC	0.846 (0.017)	0.835 (0.014)	0.838 (0.016)	0.841 (0.018)	0.787 (0.020)	0.799 (0.020)	0.802 (0.018)
36	4	AUC	0.789 (0.016)	0.755 (0.032)	0.763 (0.031)	0.764 (0.014)	0.773 (0.019)	0.786 (0.018)	0.786 (0.015)
38	4	AUC	0.753 (0.030)	0.707 (0.026)	0.709 (0.027)	0.710 (0.020)	0.719 (0.016)	0.745 (0.016)	0.745 (0.014)
40	4	AUC	0.845 (0.026)	0.890 (0.016)	0.893 (0.014)	0.900 (0.016)	0.808 (0.025)	0.825 (0.026)	0.829 (0.015)
28	4	PPV @ 90% Sensitivity	0.006 (0.001)	0.006 (0.005)	0.007 (0.005)	0.008 (0.005)	0.008 (0.000)	0.009 (0.000)	0.010 (0.003)
30	4	PPV @ 90% Sensitivity	0.012 (0.001)	0.011 (0.000)	0.012 (0.000)	0.017 (0.006)	0.016 (0.004)	0.019 (0.004)	0.019 (0.008)
32	4	PPV @ 90% Sensitivity	0.026 (0.004)	0.028 (0.007)	0.029 (0.007)	0.032 (0.011)	0.024 (0.007)	0.027 (0.007)	0.030 (0.011)
34	4	PPV @ 90% Sensitivity	0.062 (0.006)	0.071 (0.012)	0.075 (0.012)	0.076 (0.012)	0.057 (0.007)	0.061 (0.007)	0.063 (0.005)
36	4	PPV @ 90% Sensitivity	0.064 (0.004)	0.097 (0.015)	0.101 (0.014)	0.101 (0.004)	0.076 (0.005)	0.080 (0.005)	0.081 (0.006)
38	4	PPV @ 90% Sensitivity	0.039 (0.004)	0.065 (0.007)	0.067 (0.007)	0.072 (0.003)	0.043 (0.001)	0.047 (0.001)	0.048 (0.005)
40	4	PPV @ 90% Sensitivity	0.016 (0.004)	0.039 (0.010)	0.040 (0.010)	0.044 (0.005)	0.021 (0.006)	0.022 (0.006)	0.022 (0.008)
28	4	Specificity @ 90% Sensitivity	0.493 (0.111)	0.384 (0.237)	0.489 (0.246)	0.517 (0.000)	0.486 (0.000)	0.544 (0.000)	0.580 (0.120)
30	4	Specificity @ 90% Sensitivity	0.553 (0.024)	0.385 (0.000)	0.486 (0.000)	0.561 (0.174)	0.563 (0.142)	0.564 (0.145)	0.603 (0.087)
32	4	Specificity @ 90% Sensitivity	0.557 (0.068)	0.520 (0.000)	0.544 (0.000)	0.556 (0.158)	0.428 (0.116)	0.487 (0.114)	0.494 (0.141)
34	4	Specificity @ 90% Sensitivity	0.571 (0.043)	0.501 (0.077)	0.516 (0.078)	0.521 (0.081)	0.418 (0.070)	0.453 (0.067)	0.474 (0.049)
36	4	Specificity @ 90% Sensitivity	0.415 (0.035)	0.385 (0.087)	0.401 (0.083)	0.412 (0.026)	0.417 (0.034)	0.446 (0.035)	0.451 (0.041)
38	4	Specificity @ 90% Sensitivity	0.393 (0.070)	0.347 (0.076)	0.360 (0.076)	0.413 (0.024)	0.331 (0.015)	0.393 (0.015)	0.400 (0.067)
40	4	Specificity @ 90% Sensitivity	0.580 (0.094)	0.740 (0.000)	0.743 (0.000)	0.775 (0.034)	0.562 (0.102)	0.591 (0.107)	0.615 (0.000)

© 2026 Li H et al. *JAMA Network Open*.

Supplementary eTable 6. Comparison of machine learning–based models and a risk score–based model for prediction of preeclampsia (PE). This table compares the performance of the primary machine learning–based model and a conventional risk score–based model across two gestational age (GA) observation windows (up to 32 weeks and up to 38 weeks), each with a fixed 2-week prediction horizon. Model performance is reported for the development cohort (WCMC) and external validation cohorts (LMH and BMH). Area under the receiver operating characteristic curve (AUC) is shown overall. Specificity and positive predictive value (PPV) were calculated at a fixed sensitivity of 90%. Values are presented as mean (SD). Feature used in Risk score Advanced age (≥35 years), nulliparious, Black race, obesity (BMI ≥ 30 kg/m^2) and multifetal pregnancy; Model used in Risk score: XGBoost.

	**Observation window up to 32 weeks and a 2-week prediction window (32–34 weeks)**			**Observation window up to 38 weeks and a 2-week prediction window (38–40 weeks)**		
	WCMC	LMH	BMH	WCMC	LMH	BMH
	**AUC**					
**Primary analysis**	0.863 ± 0.018	0.833 ± 0.014	0.806 ± 0.016	0.756 ± 0.015	0.716 ± 0.017	0.729 ± 0.017
**Risk Score**	0.734 ± 0.020	0.707 ± 0.019	0.678 ± 0.016	0.662 ± 0.016	0.627 ± 0.014	0.622 ± 0.019
	**Specificity @ 90% Sensitivity**					
**Primary analysis**	0.569 ± 0.127	0.424 ± 0.161	0.471 ± 0.000	0.406 ± 0.046	0.327 ± 0.054	0.316 ± 0.124
**Risk Score**	0.297 ± 0.073	0.205± 0.091	0.242± 0.021	0.279 ± 0.029	0.197 ± 0.038	0.185 ± 0.029
	**PPV @ 90% Sensitivity**					
**Primary analysis**	0.009 ± 0.003	0.007 ± 0.003	0.009 ± 0.000	0.032 ± 0.002	0.053 ± 0.004	0.032 ± 0.003
**Risk Score**	0.005 ± 0.001	0.003 ± 0.001	0.004 ± 0.002	0.027 ± 0.001	0.045 ± 0.005	0.026 ± 0.002

© 2026 Li H et al. *JAMA Network Open*.

Supplementary Figure

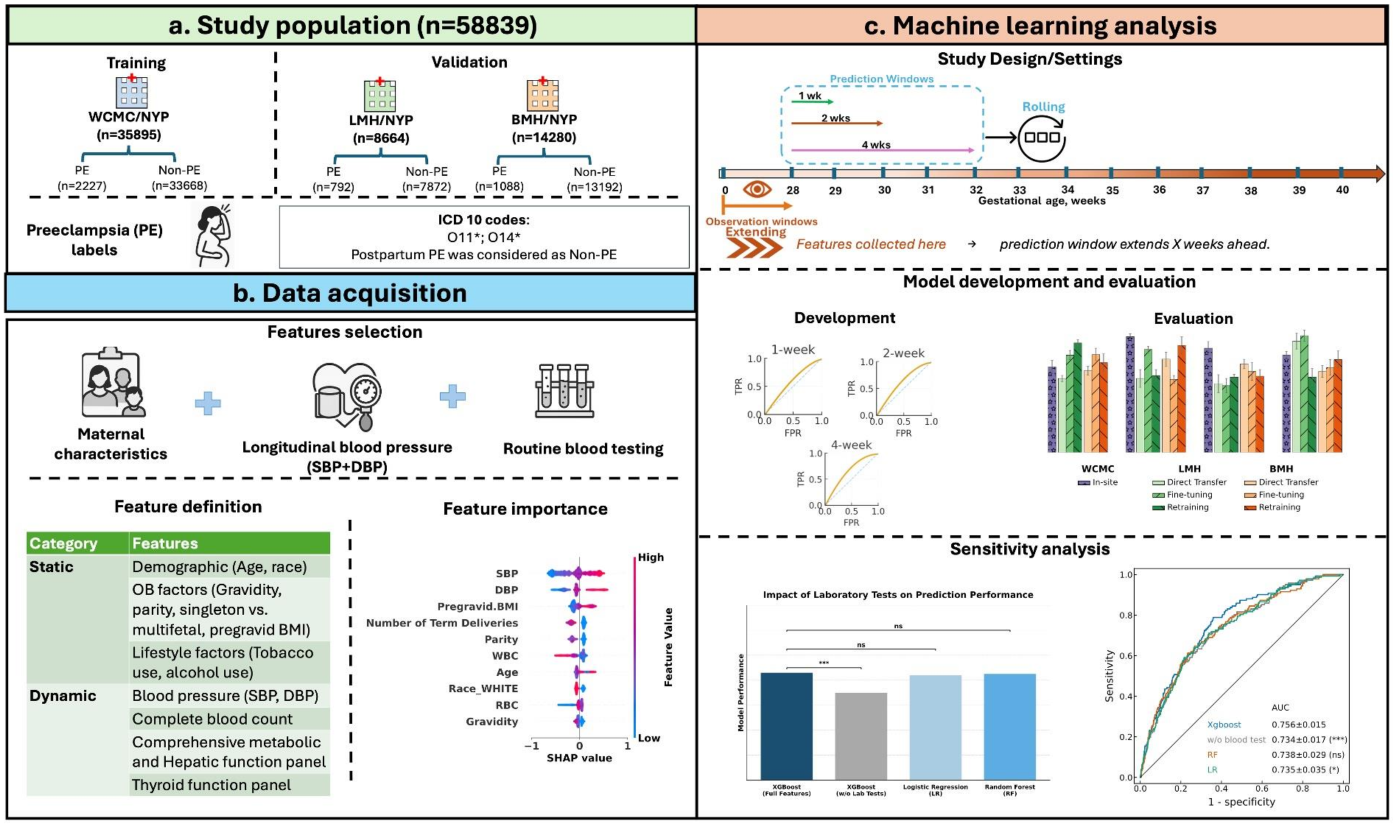

Supplementary eFigure 1: Study design and machine learning framework for preeclampsia (PE) prediction. (a) Cohorts included a training set from Weill Cornell (WC/NYP) and two external validation sets (LMH/NYP and BMH/NYP). (b) Input features comprised maternal characteristics, blood pressure, and routine laboratory panels; Feature importance were presented by SHAP value. (c) Models were developed across rolling observation windows (0–28 to 0–40 weeks) and short-term prediction horizons (1–4 weeks). XGBoost achieved the best performance compared with random forest and logistic regression, and sensitivity analyses showed that exclusion of laboratory data modestly reduced model accuracy.

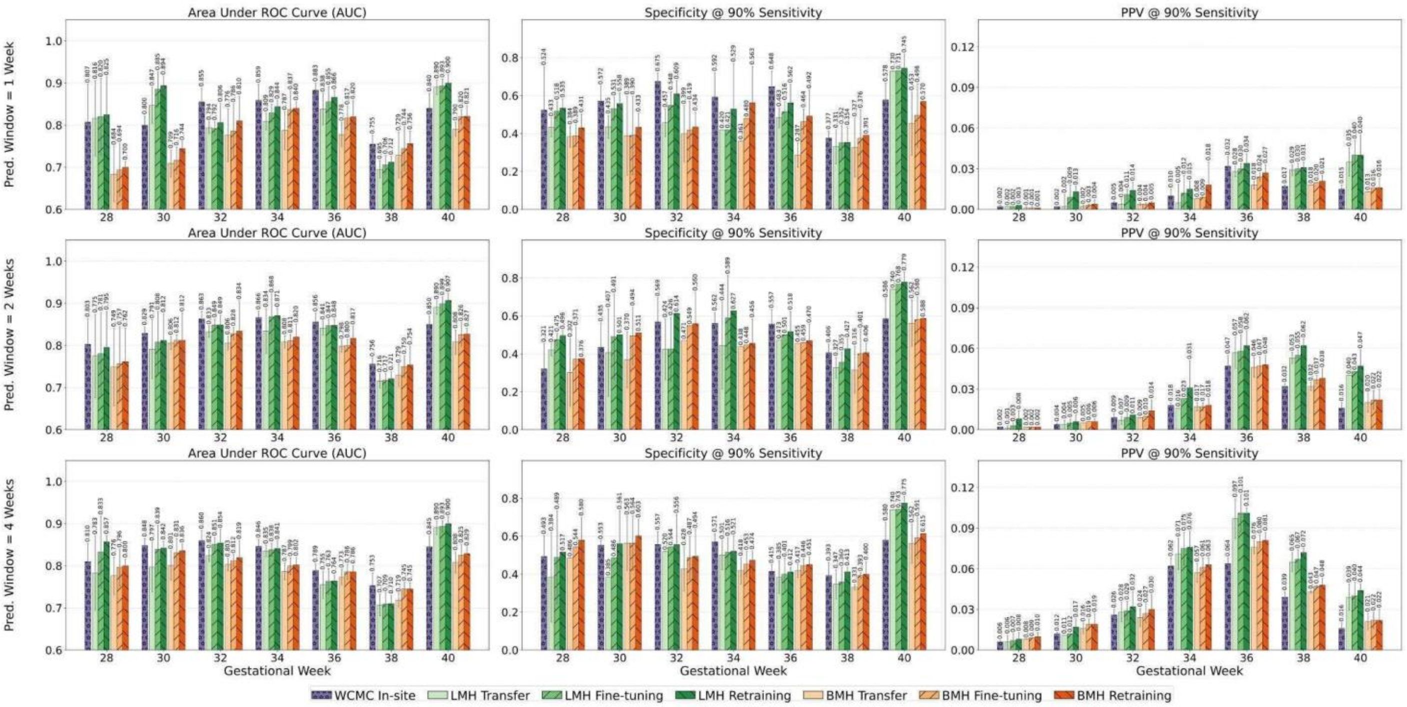

Supplementary eFigure 2: Prediction performance across gestational windows, prediction windows, and cross-site strategies. The prediction performance followed a consistent pattern across gestational windows, peaking around 34 weeks, declining at 38 weeks, then recovering at delivery across three different hospitals. Model performance was evaluated at index gestational weeks 28, 30, 32, 34, 36, and 38 with prediction windows of 1 week (top row), 2 weeks (middle row), and 4 weeks (bottom row). Each panel reports results from WCMC (in-site benchmark), LMH (external site), and BMH (external site) under three adaptation strategies: direct transfer, fine-tuning with local data, and complete model retraining. Metrics include AUC (left column), sensitivity at 90% specificity (middle column), and positive predictive value (right column). Across all sites, AUC and sensitivity improved as the observation window extended from 28 to 34 weeks and then plateaued through 38 weeks, while performance declined with longer prediction windows. Direct transfer resulted in lower external performance, whereas fine-tuning and retraining consistently restored accuracy toward in-site levels, demonstrating the value of local adaptation. AUC: Area Under the

Receiver Operating Characteristic Curve; WCMC: Weill Cornell Medical College, LMH: Lower Manhattan Hospital, BMH: Brooklyn Methodist Hospital.

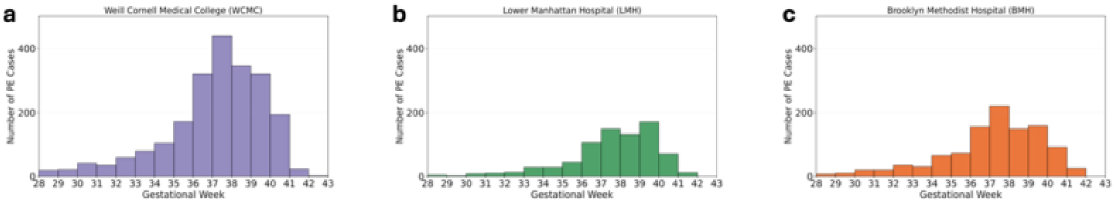

Supplementary eFigure 3. The distribution of PE patient counts across gestational weeks of three hospitals.

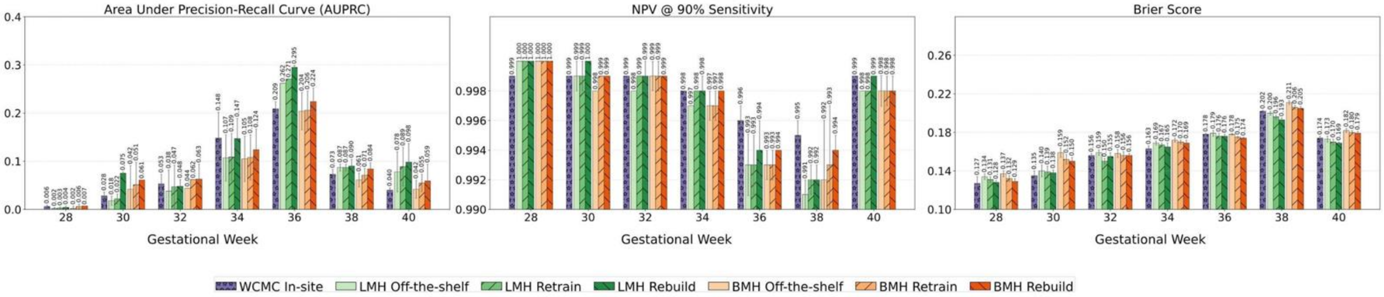

Supplementary eFigure 4. Prediction performance across gestational windows and cross-site strategies (2-week prediction window). AUPRC: Area under the precision–recall curve; WCMC: Weill Cornell Medical College, LMH: Lower Manhattan Hospital, BMH: Brooklyn Methodist Hospital.

© 2026 Li H et al. *JAMA Network Open*.

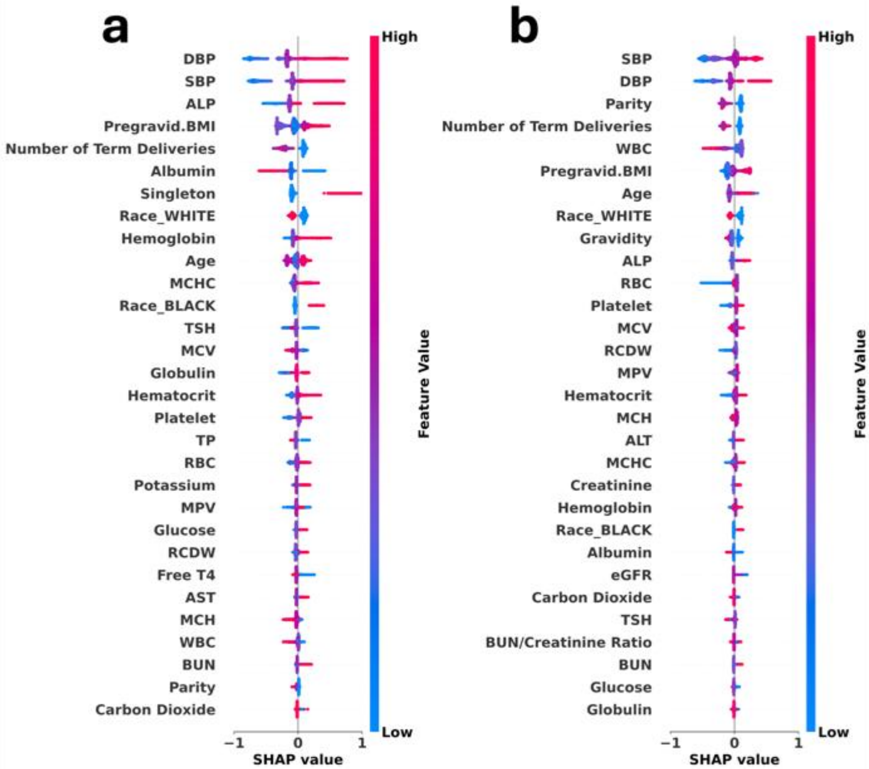

Supplementary eFigure 5. Top-30 global feature importance for PE prediction at early and late gestational stages. Global SHAP analyses were performed on XGBoost models (LMH direct transfer). (a) Observation window up to 32 weeks with a 2-week prediction window (32–34 weeks), representing early-onset prediction. (b) Observation window up to 38 weeks with a 2-week prediction window (38–40 weeks), representing late-onset prediction. In these SHAP summary plots, each dot represents an individual pregnancy and colors indicate feature values (red = high, blue = low).

© 2026 Li H et al. *JAMA Network Open*.

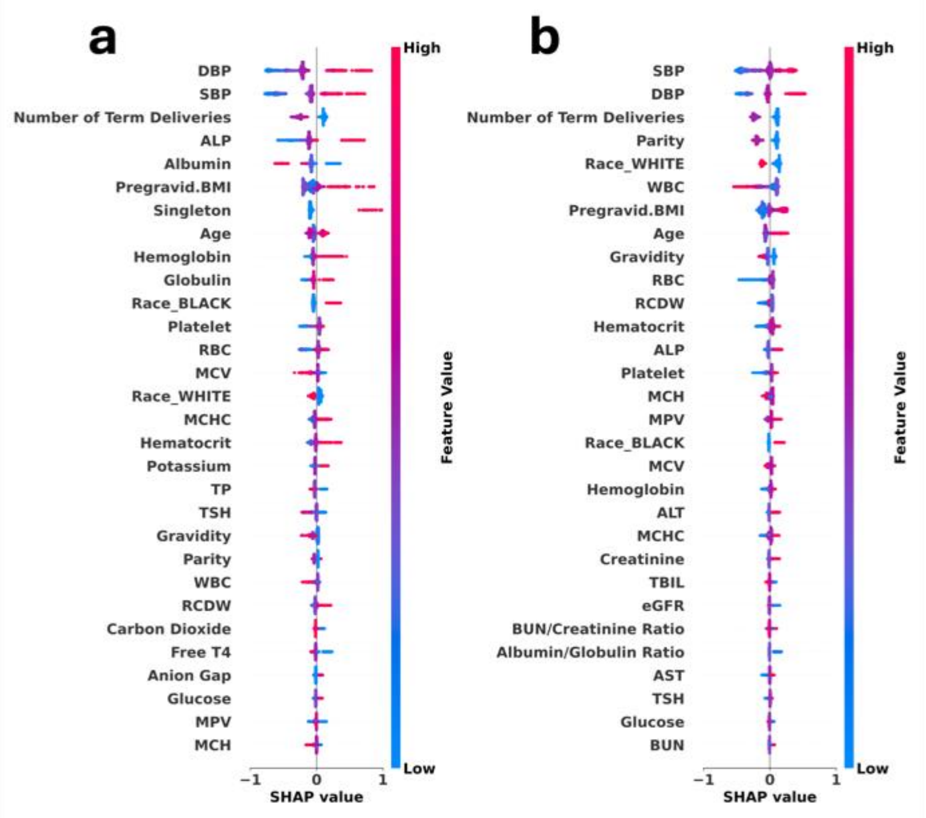

Supplementary eFigure 6. Top-30 global feature importance for PE prediction at early and late gestational stages. Global SHAP analyses were performed on XGBoost models (LMH fine-tuning). (a) Observation window up to 32 weeks with a 2-week prediction window (32–34 weeks), representing early-onset prediction. (b) Observation window up to 38 weeks with a 2-week prediction window (38–40 weeks), representing late-onset prediction. In these SHAP summary plots, each dot represents an individual pregnancy and colors indicate feature values (red = high, blue = low).

© 2026 Li H et al. *JAMA Network Open*.

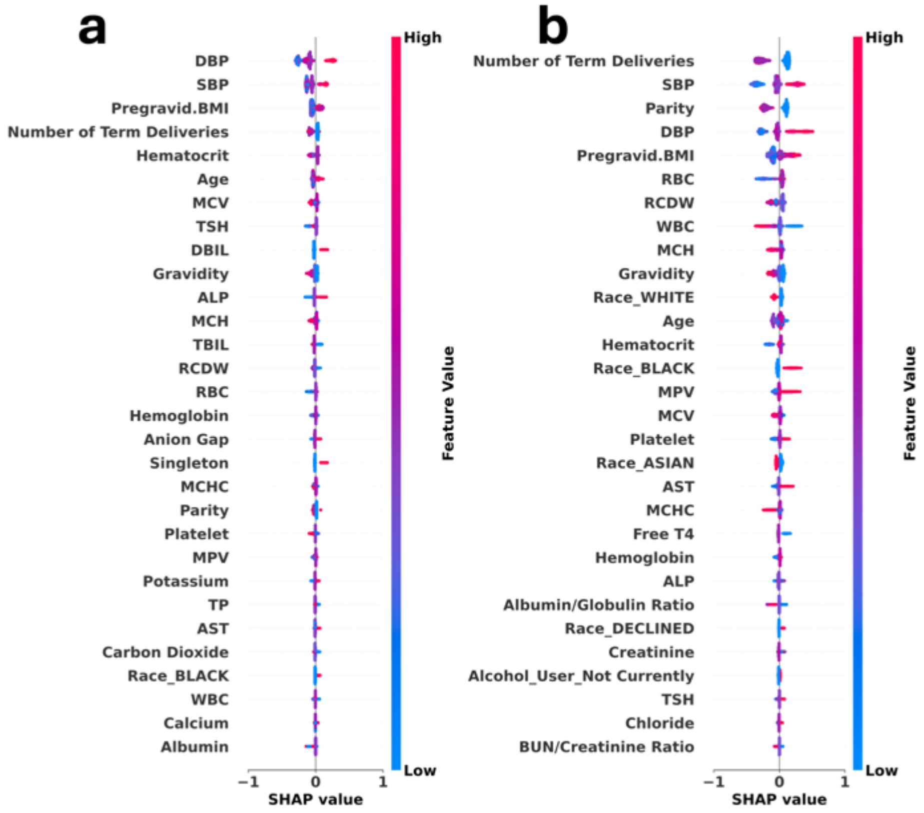

Supplementary eFigure 7. Top-30 global feature importance for PE prediction at early and late gestational stages. Global SHAP analyses were performed on XGBoost models (LMH retraining). (a) Observation window up to 32 weeks with a 2-week prediction window (32–34 weeks), representing early-onset prediction. (b) Observation window up to 38 weeks with a 2-week prediction window (38–40 weeks), representing late-onset prediction. In these SHAP summary plots, each dot represents an individual pregnancy and colors indicate feature values (red = high, blue = low).

© 2026 Li H et al. *JAMA Network Open*.

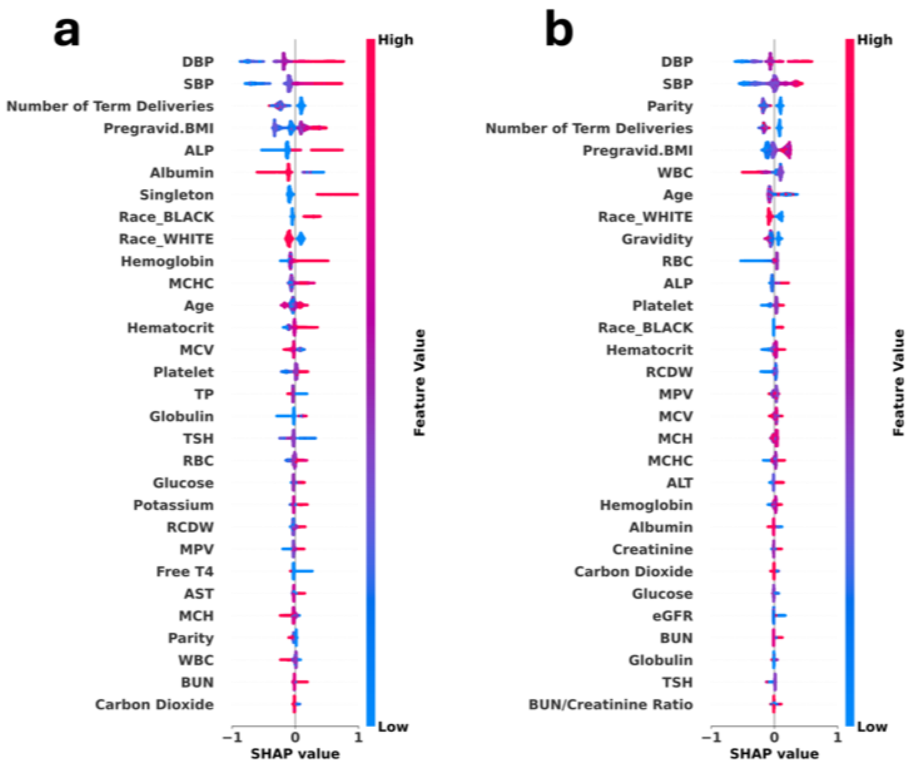

Supplementary eFigure 8. Top-30 global feature importance for PE prediction at early and late gestational stages. Global SHAP analyses were performed on XGBoost models (BMH direct transfer). (a) Observation window up to 32 weeks with a 2-week prediction window (32–34 weeks), representing early-onset prediction. (b) Observation window up to 38 weeks with a 2-week prediction window (38–40 weeks), representing late-onset prediction. In these SHAP summary plots, each dot represents an individual pregnancy and colors indicate feature values (red = high, blue = low).

© 2026 Li H et al. *JAMA Network Open*.

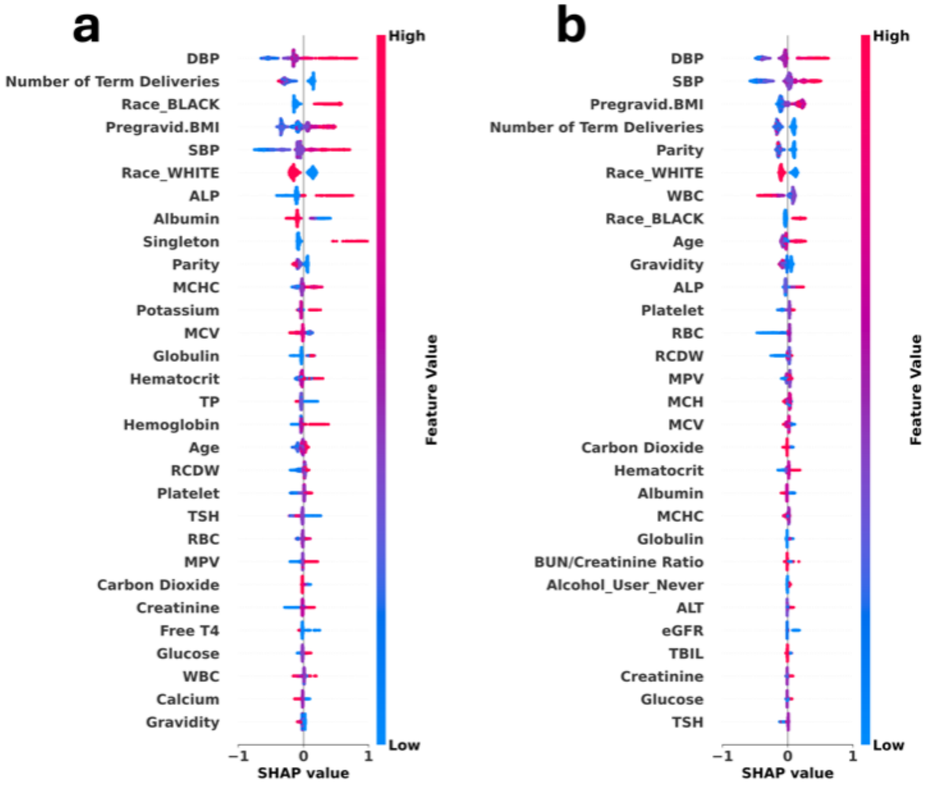

Supplementary eFigure 9. Top-30 global feature importance for PE prediction at early and late gestational stages. Global SHAP analyses were performed on XGBoost models (BMH fine-tuning). (a) Observation window up to 32 weeks with a 2-week prediction window (32–34 weeks), representing early-onset prediction. (b) Observation window up to 38 weeks with a 2-week prediction window (38–40 weeks), representing late-onset prediction. In these SHAP summary plots, each dot represents an individual pregnancy and colors indicate feature values (red = high, blue = low).

© 2026 Li H et al. *JAMA Network Open*.

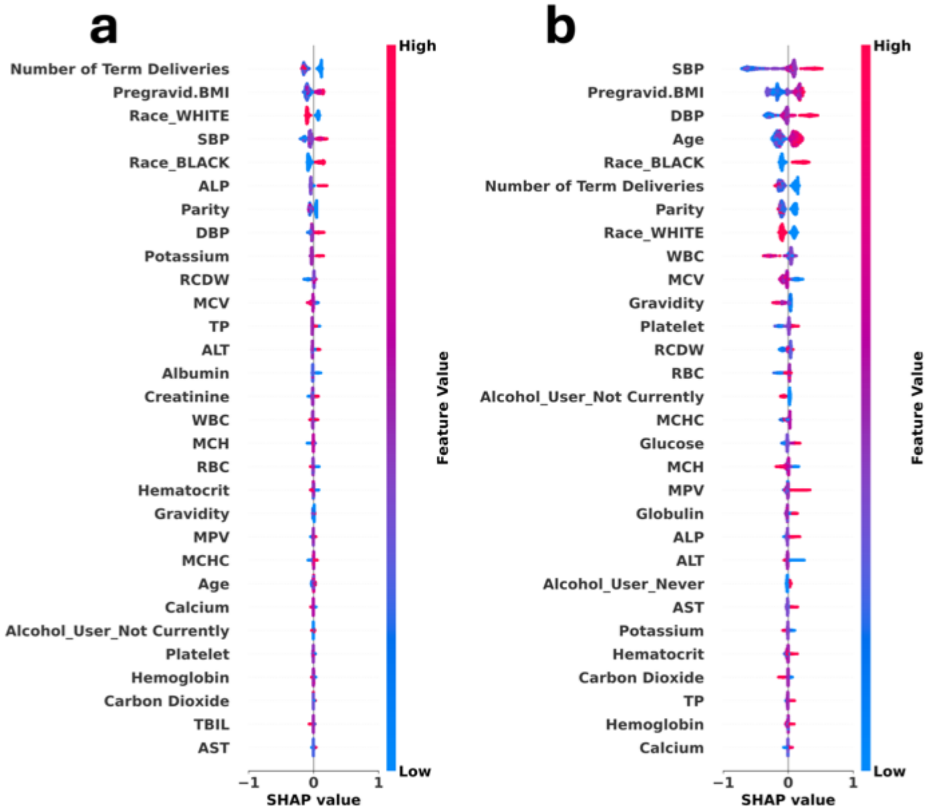

Supplementary eFigure 10. Top-30 global feature importance for PE prediction at early and late gestational stages. Global SHAP analyses were performed on XGBoost models (BMH retraining). (a) Observation window up to 32 weeks with a 2-week prediction window (32–34 weeks), representing early-onset prediction. (b) Observation window up to 38 weeks with a 2-week prediction window (38–40 weeks), representing late-onset prediction. In these SHAP summary plots, each dot represents an individual pregnancy and colors indicate feature values (red = high, blue = low).

© 2026 Li H et al. *JAMA Network Open*.

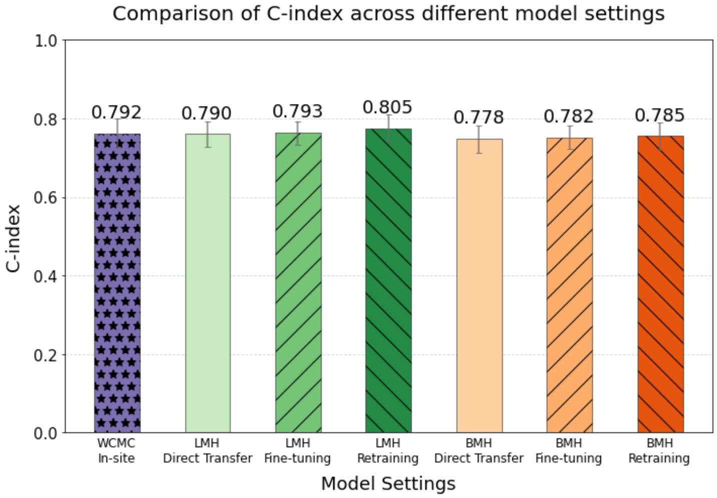

Supplementary eFigure 11. Comparison of discrimination performance between the primary binary prediction framework and a Cox proportional hazards model across different settings. This figure reports the concordance index (C-index) of a Cox proportional hazards model evaluated under the same data splits and site-specific deployment settings as the primary analysis, including in-site evaluation at WCMC and external validation at LMH and BMH with off-the-shelf, retrained, and rebuilt model configurations.

© 2026 Li H et al. *JAMA Network Open*.

eMethods

Data and cohort

This study was approved by the Institutional Review Board of Weill Cornell Medicine (IRB#24-05027450). All analyses were performed on de-identified EHR data. The study was approved by the Weill Cornell Medicine Institutional Review Board with a waiver of informed consent for secondary use of de-identified EHR data. Reporting of the results adhered to the Strengthening the Reporting of Observational studies in Epidemiology (STROBE) and Transparent Reporting of a multivariable prediction model for individual Prognosis Or Diagnosis + Artificial Intelligence (TRIPOD+AI) reporting guidelines [6].

We conducted a retrospective, multi-site cohort study of pregnant individuals who received prenatal care and delivered at three New York Presbyterian hospitals: Weill Cornell Medical College (WCMC), Lower Manhattan Hospital (LMH), and Brooklyn Methodist Hospital (BMH). The dataset includes structured clinical information spanning demographics, vital signs, laboratory results, and diagnosis codes, recorded as part of routine obstetric care. The study cohort was constructed by identifying pregnancies with a documented delivery date between October 2020 and May 2025. Inclusion criteria required patients to be 18 years or older with documented delivery records that included both delivery time and gestational age (GA) at delivery during the study period. After excluding 247 pregnancies (142 from WCMC, 22 from LMH, and 83 from BMH) due to missing delivery time or GA at delivery, the final cohort comprised 58,839 pregnancies: 35,895 from WCMC, 8,664 from LMH, and 14,280 from BMH. Each pregnancy was treated as a distinct observation. Multiple gestations were included, with no restrictions on parity. GA across the pregnancy was calculated based on the delivery date and delivery GA recorded in the EHR. Clinical variables were aligned to gestational week using date of service and delivery date/GA-

© 2026 Li H et al. *JAMA Network Open*.

derived reference timepoints. Pregnancies were identified by a clinician-documented delivery encounter; pregnancy onset (GA = 0 weeks, LMP-equivalent) was back-calculated as delivery date minus GA at delivery, and delivery GA/date/time were abstracted from the delivery record. PE was defined using ICD-10 diagnostic codes corresponding to antepartum PE, including both mild and severe subtypes (O11.x and O14.x). The time of PE onset was operationalized as the admission date of the first diagnosis code within the pregnancy episode. Vital signs and laboratory tests were retrieved from structured flowsheets and laboratory panels, respectively. Data available for model development included demographics (maternal age, race), obstetric characteristics (parity, gravidity, pregravid BMI, singleton vs. multifetal gestation), vital signs (blood pressure), lifestyle (tobacco or alcohol use) and laboratory results including complete blood count (CBC) panel, comprehensive metabolic panel (CMP), Hepatic and Thyroid functions. All features were time-stamped.

Feature extraction and preprocessing

We summarized features used for model development in Supplementary Table 1. Demographic and obstetric characteristics, including maternal age, race, gravidity, parity, pregravid BMI, and pregnancy type (singleton vs. multifetal), were treated as fixed covariates, with lifestyle factors (tobacco/alcohol use) also incorporated. Blood pressure features were summarized as the median of all prenatal systolic and diastolic measurements within the observation window (defined in the Experimental Setting), minimizing the impact of outliers while reflecting overall maternal hemodynamics. For laboratory analytes, the most recent value within the observation window was used to capture the latest clinical status. Input variables were selected a priori based on clinical relevance, routine availability in prenatal care, and prior evidence of association with PE. Demographic and obstetric characteristics reflect established baseline risk factors, blood pressure

© 2026 Li H et al. *JAMA Network Open*.

captures evolving maternal hemodynamics, and laboratory tests provide objective measures of systemic, hepatic, renal, and hematologic changes preceding clinical diagnosis. Variables requiring specialized testing or limited availability were intentionally excluded to enhance scalability and real-world applicability. Categorical variables were one-hot encoded, while continuous features were imputed with the median and categorical features with the mode, estimated within each training fold to prevent leakage. Continuous variables were z-standardized using statistics from the training split, with the same transformation applied to validation and test sets [7]. Finally, the modeling features comprised the most recent laboratory results, median systolic and diastolic blood pressure (SBP and DBP), and static demographic, obstetric, and lifestyle variables including tobacco use and alcohol use.

Experimental setting

We formulated the task as a binary classification problem anchored to GA. For each pregnant individual, we defined two key temporal intervals: (1) Observation window: the period from conception up to a cutoff week t_{obs}, during which clinical and laboratory data are collected as inputs. (2) Prediction window: a future time interval of length Δ weeks, spanning $[t_{\text{obs}}, t_{\text{obs}} + \Delta]$, during which the model aims to forecast whether PE will occur. Let T denote GA in weeks. The objective is to predict, at time t_{obs}, whether a patient will develop PE during the subsequent prediction window. Patients who develop PE in $[t_{\text{obs}}, t_{\text{obs}} + \Delta]$ are labeled as positive; those who do not develop PE by the end of the prediction window are labeled as negative. Formally, let $\mathbf{x}_{\boldsymbol{i}}^{(\boldsymbol{t}_{\text{obs}})}$ represent the feature vector for the individual i, constructed from all available data up to t_{obs}. The outcome variable is defined as:

$$y_i^{(t_{\text{obs}})} = \begin{cases} 1, & \text{if PE onset occurs in } [t_{\text{obs}}, t_{\text{obs}} + \Delta] \\ 0, & \text{otherwise} \end{cases}. \quad (1)$$

© 2026 Li H et al. *JAMA Network Open*.

We then trained a probabilistic binary classifier f to estimate the risk score for each patient:

$$\hat{y}_i^{(t_{\text{obs}})} = f\left(\mathbf{x}_i^{(t_{\text{obs}})}\right), \quad \hat{y}_i^{(t_{\text{obs}})} \in [0,1]. \tag{2}$$

Model development and validation

We implemented extreme gradient boosting (XGBoost) as the primary modeling framework [8]. All experiments followed a nested cross-validation design, with both the outer and inner loops based on stratified 5-fold splits [9]. The outer loop consisted of 5-fold cross-validation, where the dataset was split into five patient-level folds. In each round, one-fold was held out for evaluation, and the remaining four folds were used for training and hyperparameter tuning. The inner loop used a 5-fold split on the training data to identify optimal hyperparameters via grid search on the validation set. The final reported metrics were averaged across the five outer test folds. All preprocessing steps, including imputation, feature scaling, and model training, were performed within each training fold to avoid information leakage [10]. The chosen hyperparameters from validation were then applied to retrain the model on the full training data before evaluation on the outer test fold. Because the incidence of PE was relatively low, we applied sample reweighting to balance positive and negative classes with the class-weighted loss functions in XGBoost, where the positive class weight was set proportional to the inverse prevalence [11]:

$$\text{weight}_{\text{PE}} = \frac{n_{\text{neg}}}{n_{\text{pos}}}, \tag{3}$$

with n_{pos} and n_{neg} denoting the number of positive and negative samples in training folds, respectively. In sensitivity analyses, we compared other machine-learning prediction models, including regularized logistic regression and random forests, [12,13] under the same experimental setup as the primary analysis, enabling direct comparisons.

© 2026 Li H et al. *JAMA Network Open*.

Cross-site evaluation strategies

To evaluate generalizability across healthcare systems, we designed four complementary strategies for model transfer and adaptation [14-16]. (1) **In-site testing**: Models were trained and validated within the same hospital using nested cross-validation; this was applied only at WCMC, which served as the internal performance benchmark. (2) **Direct transfer** [17]: Models trained at WCMC, where the largest number of pregnancies was available, were directly applied to LMH and BMH without modification. This represented the simplest form of external validation but was expected to be most sensitive to distributional differences in demographics, laboratory availability, and clinical practice patterns. (3) **Fine-tuning**: The model architecture and hyperparameters tuned at WCMC were fixed, with additional trees added to the existing XGBoost model using site-specific training data from LMH or BMH. This preserved the source model's structure while adapting to local feature distributions, requiring moderate computational and data resources. (4) **Retraining**: Complete model redevelopment was performed separately at LMH and BMH, including new hyperparameter optimization within each site. This maximized alignment to local data but required the greatest resources and reduced cross-site comparability. Performance under these strategies was assessed for each observation window and prediction window. Overall, in-site testing reflected best-case accuracy within a given site, direct transfer represented external portability without adaptation, and fine-tuning or retraining quantified the benefits of local adaptation.

Statistical analysis

Feature importance analysis

To interpret model predictions, we applied SHapley Additive exPlanations (SHAP) to the trained XGBoost models [18]. SHAP values were calculated for each feature in the outer test folds of the nested cross-validation pipeline, ensuring that attribution was based only on held-out data. Each

© 2026 Li H et al. *JAMA Network Open*.

point in the SHAP beeswarm plot reflects the contribution of a feature for a pregnancy, allowing pregnancy-level ("local") interpretation in addition to the global importance ranking. Global feature importance was summarized using the mean absolute SHAP value across all individuals. We examined feature contributions separately for early-onset prediction and late-onset prediction. Early-onset prediction was represented by an observation window up to 32 weeks with a 2-week prediction window (32–34 weeks), while late-onset prediction was represented by an observation window up to 38 weeks with a 2-week prediction window (38–40 weeks). This design allowed us to assess whether the importance of specific features shifted between earlier and later stages of pregnancy. The feature set included demographics (e.g., age, race/ethnicity), obstetric history (e.g., gravidity, parity, pregravid BMI, pregnancy type), lifestyle factors (tobacco and alcohol use), vital signs (median SBP and DBP), and laboratory tests (CBC panel, CMP, Hepatic and Thyroid functions), see Supplemental Table 1. For each model, the ranked feature list highlighted both clinical covariates and laboratory markers that contributed most strongly to predictions of PE risk. Results from SHAP analyses were visualized to compare the global feature rankings between early and late prediction tasks. These comparisons enabled identification of features with consistent predictive value across gestation, as well as features whose contributions were stage-specific.

Evaluation metrics

Model performance was quantified using several complementary metrics: (1) Area under the receiver operating characteristic curve (AUC) [19]. AUC measured overall discrimination between pregnancies that did and did not develop PE within the prediction window. (2) Area under the precision–recall curve (AUPRC) [19]. AUPRC summarized the trade-off between precision and recall across decision thresholds and is particularly informative for imbalanced outcomes such as PE, where positive cases are relatively rare. (3) Specificity at 90% sensitivity [16,20]: To reflect the clinical

© 2026 Li H et al. *JAMA Network Open*.

priority of minimizing missed cases, we fixed the operating point at 90% sensitivity and reported the corresponding specificity, capturing the ability to detect true PE cases while limiting false positives to 10% of unaffected pregnancies. Thresholds were determined within the inner cross-validation loop and applied to the corresponding outer test fold. (4) Positive predictive value (PPV) and negative predictive value (NPV) at 90% sensitivity [21]: PPV measured the proportion of true PE cases among pregnancies classified as high risk, while NPV measured the proportion of true non-PE pregnancies among those classified as low risk. Because PE prevalence is relatively low, PPV served as a critical indicator of clinical utility beyond overall discrimination. (5) Brier score. The Brier score measured the mean squared error between predicted probabilities and observed outcomes, jointly reflecting discrimination and calibration, with lower values indicating better calibrated and more accurate probabilistic predictions.

Performance was summarized at each gestational index week and for each prediction window (1, 2, and 4 weeks). These metrics were reported consistently across in-site testing, direct transfer, fine-tuning, and retraining strategies, enabling direct comparison of discrimination, detection capability under fixed specificity, and precision across sites.

Sensitivity analyses

To examine the robustness of our findings, we performed a series of sensitivity analyses. First, we evaluated the impact of excluding laboratory tests or blood pressure measurements from the feature set and only including blood pressure measurements in the feature set. Second, we compared XGBoost with two baseline classifiers: logistic regression and random forest. Both models were trained and validated on the same cross-validation folds and feature sets as XGBoost. Performance under these sensitivity settings was assessed using the same evaluation metrics.

© 2026 Li H et al. *JAMA Network Open*.

Third, we also examined additional analytical choices, including deep learning–based models [1-5], patient-level evaluation, and feature exclusion based on missingness.

Survival analyses

We additionally implemented a Cox proportional hazards model, with time-to-event defined as GA at PE onset and censoring at delivery for pregnancies without the outcome [22]. Model discrimination was evaluated using the concordance index (C-index) on the same in-site and external validation cohorts and deployment settings as the primary analysis.

© 2026 Li H et al. *JAMA Network Open*.

References

1. Hollmann N, Muller S, Purucker L, et al. Accurate predictions on small data with a tabular foundation model. *Nature*. Jan 2025;637(8045):319-326. doi:10.1038/s41586-024-08328-6
2. Gorishniy Y, Kotelnikov A, Babenko A. Tabm: Advancing tabular deep learning with parameter-efficient ensembling. *arXiv preprint arXiv:241024210*. 2024;
3. Huang X, Khetan A, Cvitkovic M, Karnin Z. Tabtransformer: Tabular data modeling using contextual embeddings. *arXiv preprint arXiv:201206678*. 2020;
4. Somepalli G, Goldblum M, Schwarzschild A, Bruss CB, Goldstein T. Saint: Improved neural networks for tabular data via row attention and contrastive pre-training. *arXiv preprint arXiv:210601342*. 2021;
5. Song W, Shi C, Xiao Z, et al. Autoint: Automatic feature interaction learning via self-attentive neural networks. 2019:1161-1170.
6. Cuschieri S. The STROBE guidelines. *Saudi J Anaesth*. Apr 2019;13(Suppl 1):S31-S34. doi:10.4103/sja.SJA_543_18
7. Milligan GW, Cooper MC. A study of standardization of variables in cluster analysis. *Journal of classification*. 1988;5(2):181-204.
8. Chen T, Guestrin C. Xgboost: A scalable tree boosting system. 2016:785-794.
9. Berrar D. Cross-validation. 2019.
10. Song C, Raghunathan A. Information leakage in embedding models. 2020:377-390.
11. Kalton G, Flores-Cervantes I. Weighting methods. *Journal of official statistics*. 2003;19(2):81.
12. LaValley MP. Logistic regression. *Circulation*. 2008;117(18):2395-2399.
13. Breiman L. Random forests. *Machine learning*. 2001;45(1):5-32.
14. Song X, Yu ASL, Kellum JA, et al. Cross-site transportability of an explainable artificial intelligence model for acute kidney injury prediction. *Nat Commun*. Nov 9 2020;11(1):5668. doi:10.1038/s41467-020-19551-w
15. Wu Y, Dobriban E, Davidson S. Deltagrad: Rapid retraining of machine learning models. PMLR; 2020:10355-10366.
16. Zang C, Hou Y, Lyu D, et al. Accuracy and transportability of machine learning models for adolescent suicide prediction with longitudinal clinical records. *Transl Psychiatry*. Jul 31 2024;14(1):316. doi:10.1038/s41398-024-03034-3
17. Zhuang F, Qi Z, Duan K, et al. A comprehensive survey on transfer learning. *Proceedings of the IEEE*. 2020;109(1):43-76.
18. Lundberg SM, Lee S-I. A unified approach to interpreting model predictions. *Advances in neural information processing systems*. 2017;30
19. Bradley AP. The use of the area under the ROC curve in the evaluation of machine learning algorithms. *Pattern recognition*. 1997;30(7):1145-1159.
20. Dziak JJ, Coffman DL, Lanza ST, Li R, Jermiin LS. Sensitivity and specificity of information criteria. *Brief Bioinform*. Mar 23 2020;21(2):553-565. doi:10.1093/bib/bbz016
21. Wong HB, Lim GH. Measures of diagnostic accuracy: sensitivity, specificity, PPV and NPV. *Proceedings of Singapore healthcare*. 2011;20(4):316-318.
22. Therneau TM, Grambsch PM, SpringerLink. *Modeling Survival Data: Extending the Cox Model*. 1st 2000. ed. Statistics for biology and health. Springer New York : Imprint: Springer; 2000.

© 2026 Li H et al. *JAMA Network Open*.
