# Supplementary material for: Machine Learning for Dynamic and Short-Term Prediction of Preeclampsia Using Routine Clinical Data
Source: JAMA Netw Open. 2026 Mar 6;9(3):e260359. doi: 10.1001/jamanetworkopen.2026.0359 (PMC12966928; doi:10.1001/jamanetworkopen.2026.0359)
Supplement: Supplement 2. — Data Sharing Statement [file jamanetwopen-e260359-s002.pdf]

Data Sharing Statement

Li. Machine Learning for Dynamic and Short-Term Prediction of Preeclampsia Using Routine Clinical Data. *JAMA Netw Open*. Published March 03, 2026.
doi:10.1001/jamanetworkopen.2026.0359

Data
Data available: No

Additional Information
Explanation for why data not available: This study analyzed de-identified electronic health record data from three hospitals within the NewYork-Presbyterian health system. Owing to patient privacy and institutional regulations, these data are not publicly available now. Access might be provided to qualified researchers upon reasonable request to the corresponding author and subject to approval by the Weill Cornell Medicine Institutional Review Board.
